# Zinc Nitrate Hexahydrate Pseudobinary Eutectics for Near-Room-Temperature Thermal Energy Storage

**DOI:** 10.1021/acsaenm.3c00444

**Published:** 2023-12-20

**Authors:** Sophia Ahmed, Denali Ibbotson, Chase Somodi, Patrick J. Shamberger

**Affiliations:** † Department of Materials Science and Engineering, Texas A&M University, College Station, Texas 77843, United States

**Keywords:** phase change materials, salt hydrate, eutectic, thermophysical properties, thermal cycling, nucleation

## Abstract

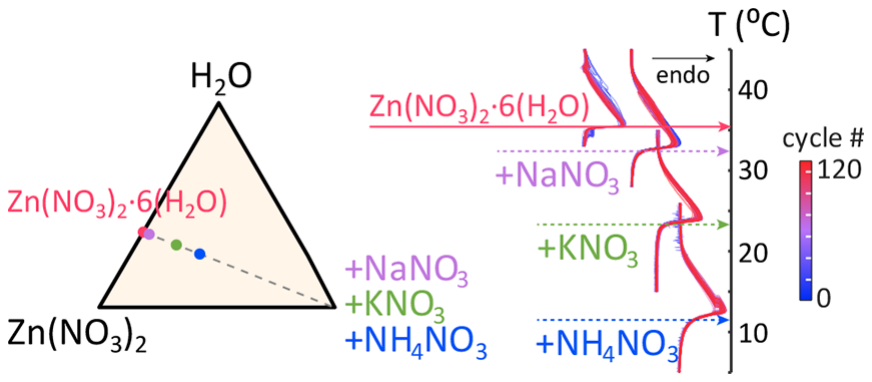

Stoichiometric salt hydrates can be inexpensive and provide higher volumetric energy density relative to other near-room-temperature phase change materials (PCMs), but few salt hydrates exhibit congruent melting behavior between 0 and 30 °C. Eutectic salt hydrates offer a strategy to design bespoke PCMs with tailored application-specific eutectic melting temperatures. However, the general solidification behavior and stability of eutectic salt hydrate systems remain unclear, as metastable solidification in eutectic salt hydrates may introduce opportunities for phase segregation. Here, we present a new family of low-cost zinc-nitrate-hexahydrate-based eutectics: Zn(NO_3_)_2_·6(H_2_O)-NaNO_3_ (*T*_eu_ = 32.7 ± 0.3 °C; *ΔH*_eu_ = 151 ± 6 J·g^–1^), Zn(NO_3_)_2_·6(H_2_O)-KNO_3_ (*T*_eu_ = 22.1 ± 0.3 °C; *ΔH*_eu_ = 140 ± 6 J·g^–1^), Zn(NO_3_)_2_·6(H_2_O)-NH_4_NO_3_ (*T*_eu_ = 11.2 ± 0.3 °C; *ΔH*_eu_ = 137 ± 5 J·g^–1^). While the tendency to undercool varies greatly between different eutectics in the family, the geologic mineral talc has been identified as an active and stable phase that dramatically reduces undercooling in Zn(NO_3_)_2_·6(H_2_O) and all related eutectics. Zn(NO_3_)_2_·6(H_2_O) and its related eutectics have shown stability for over a hundred thermal cycles in mL scale volumes, suggesting that they are capable of serving as robust and stable media for near-room-temperature thermal energy storage applications in buildings.

## 1 Introduction

**Figure 1:**
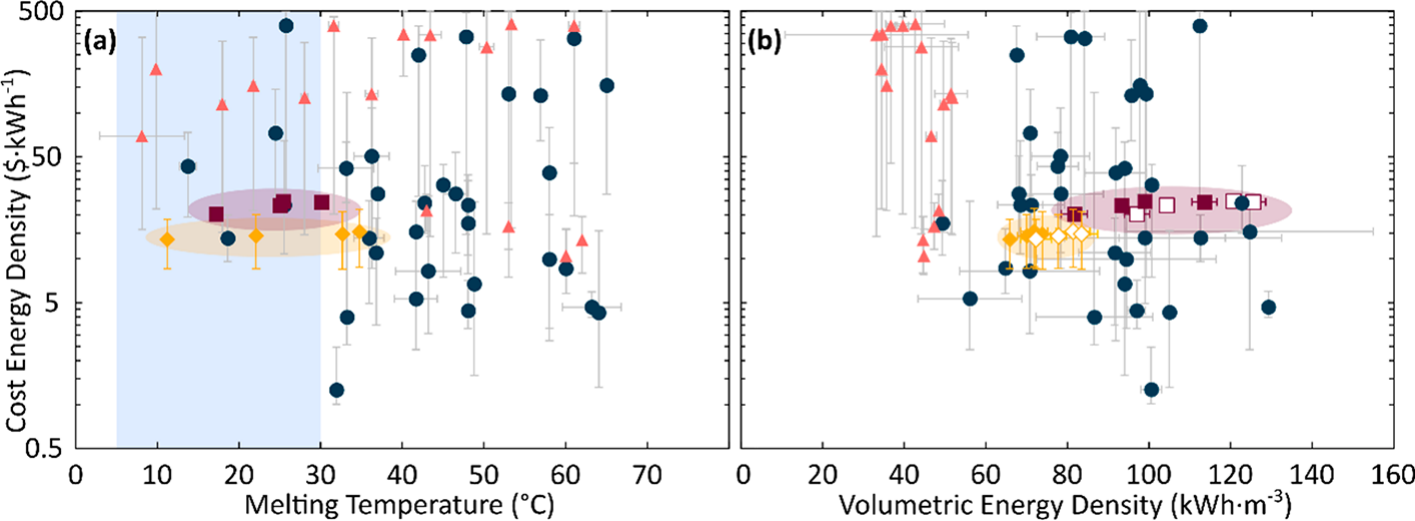
Cost-energy density as a function of (a) melting temperature of the PCM and (b) volumetric energy density of the PCM with reference data taken from Hirschey et al. and Ahmed et al.^7,8^ Figure adapted with permission from ref (7). Copyright 2021, retained by authors. Solid shapes represent salt hydrates in the liquid phase, and outlined represent the solid phase. Yellow diamonds are Zn(NO_3_)_2_·6H_2_O eutectics (reported here), maroon squares are LiNO_3_·3(H_2_O) eutectics,^8^ pink triangles are paraffin, and blue circles are other salt hydrates.^2^ The blue shaded section in (a) indicates the desired temperature range for TES media in buildings.

The use of thermal energy storage (TES) media in buildings can help to improve building energy efficiency while also reducing the carbon intensity of the domestic power grid. However, this application requires TES media that is both inexpensive and volumetrically energy dense, and which stores thermal energy at temperatures between 5 to 30 °C, compatible with most building air conditioning and heat pump systems.^1,2^ Phase change materials (PCMs) excel as TES media for use in buildings due to their high quantities of latent heat released upon phase transformations, but their usage is hindered by a lack of stable PCMs available at specific temperatures of interest for near-room-temperature thermal energy storage. Among PCMs, salt hydrates are of particular interest for building thermal storage applications due to their relatively low cost per unit energy stored (Figure 1) and nonflammable behavior.^3^ Furthermore, salt hydrates are well-known to form eutectics with other salt hydrates and anhydrous salts, thereby increasing the palette of available room-temperature PCMs.^4,5^ However, the long-term stability of these eutectics remains in question due to their metastability and the potential for metastable systems to be subject to phase segregation.^6^ In particular, both the nucleation behavior of relevant salt hydrate eutectics and the melting behavior after substantial cycling remain relatively poorly understood in eutectic systems.

TES media for use in building thermal management applications must store heat in the 5 to 30 °C temperature range depending on whether it is used as a passive storage element, or integrated into heating ventilation and air conditioning (HVAC) systems, a range in which relatively few low-cost stoichiometric salt hydrates are known to melt (Figure 1).^1^ As examples, air conditioners generally target a thermal energy storage temperature of 5 to 15 °C,^9^ and passive solar heating applications may have narrow ranges specified for their intended climate, which generally fall in the 15 to 27 °C temperature range.^10−13^ A limited number of PCMs lie within these small temperature ranges while storing a relatively large quantity of heat at a low cost (Figure 1a) and a high volumetric energy density (Figure 1b).^7^ Salt hydrates can potentially expand the number of PCMs available in these temperature ranges by forming eutectic systems. In eutectics, the resultant mixture of two or more chemical components will have a single invariant solid–liquid transformation temperature that is lower than the transformation temperature of each of its components. The selection of specific components results in control over the transformation temperature. However, only a handful of eutectic salt hydrate systems are well-established in the literature.

Salt hydrate eutectics have been explored experimentally in nitrate, chloride, and bromide hydrate systems. Nitrate-based salt hydrate eutectics have been the most widely reported, with eutectics being formed from combinations of LiNO_3_·3(H_2_O), Mg(NO_3_)_2_·6(H_2_O), Mn(NO_3_)_2_·6(H_2_O), Ca(NO_3_)_2_·4(H_2_O), and Zn(NO_3_)_2_·6(H_2_O) and with anhydrous nitrates.^5,8,9,14−16^ For example, LiNO_3_·3(H_2_O) forms both pseudobinary and ternary eutectics with anhydrous nitrates (NaNO_3_) and with other salt hydrates (Mg(NO_3_)_2_·6(H_2_O) and Zn(NO_3_)_2_·6(H_2_O)), which result in depressing the eutectic temperature by 5 to 17 °C relative to stoichiometric melting point of LiNO_3_·3(H_2_O).^8^ Similarly, Mg(NO_3_)_2_·6(H_2_O), can form eutectics with Zn(NO_3_)_2_·6H_2_O and Ca(NO_3_)_2_·4(H_2_O) to have eutectic temperatures 56 to 58 °C lower than that of Mg(NO_3_)_2_·6(H_2_O).^9^ Chloride eutectics have not been as widely explored, in part because many chloride systems are subject to incongruent melting at peritectic points, but a few have been experimentally evaluated including the CaCl_2_·6(H_2_O)-MgCl_2_·6(H_2_O) pseudobinary systems and CaCl_2_-NaCl_2_-KCl-H_2_O eutectics.^17,18^ Mixing of nitrate and chloride salt hydrates has been investigated previously, including the Mg(NO_3_)_2_·6(H_2_O)-MgCl_2_·6(H_2_O) and Mg(NO_3_)_2_·6(H_2_O)-MgBr_2_·6(H_2_O) pseudobinary systems, with melting temperatures of 59.1 and 65.85 °C respectively.^19^ The eutectic temperatures are a suppression of its components’ melting temperatures, where Mg(NO_3_)_2_·6(H_2_O), MgCl_2_·6(H_2_O), and MgBr_2_·6(H_2_O) melt at 88, 117, and 164.4 °C.^3,20^ While some salt hydrates are subject to corrosion concerns (in particular chlorides) or toxicity concerns (in particular bromides), these secondary concerns very greatly vary from system to system, and it is difficult to make general claims about these limitations across all salt hydrates.

Experimental determination of eutectic compositions can be guided by models that provide predicted eutectic temperatures and compositions from a limited set of training data. The work presented in this manuscript is based in part on computational predictions from a prior work.^4^ While these CALPHAD-type models exist they remain largely empirical and have known limitations, including the need for somewhat complicated parametrizations in some cases.^21^ The modified Brunauer–Emmett–Teller (BET) method is one of the more commonly applied approaches for calculating the activity of water in salt hydrate systems. This approach assumes that the water component and the molten salt component are considered to be reference states; excess enthalpy only considers the energy of hydration for the salts, and all salt hydrate mixtures are ideal mixtures.^4^ This can lead to discrepancies in the melting temperature or composition between the experimental values and the predictions. Previous studies concerning the modified BET model investigated the LiNO_3_·3(H_2_O)-LiClO_4_·3(H_2_O) pseudobinary eutectic, the NH_4_NO_3_-Mg(NO_3_)_2_·6(H_2_O)-Mn(NO_3_)·6(H_2_O) pseudoternary, with the former having a 1.3 °C difference between the model and experimental melting temperatures, and the latter having a −1.5 °C difference.^16^ Lithium nitrate trihydrate eutectics, LiNO_3_·3(H_2_O)-LiNO_3_-NaNO_3_, LiNO_3_·3(H_2_O)-LiNO_3_-Mg(NO_3_)_2_·6(H_2_O), LiNO_3_·3(H_2_O)-LiNO_3_-Zn(NO_3_)_2_·6(H_2_O) had absolute differences in the predicted and eutectic compositions of 0.1, 11.3, and 22.4 wt %, respectively, which significantly impact the PCM selection criteria including cost density calculations.^8^ Similarly, discrepancies with predicted eutectic temperatures, even if they are small, can be critical due to the narrow operational temperature window of some thermal energy storage components and systems. Relevant to the study presented here, a Zn(NO_3_)_2_·6(H_2_O) and anhydrous nitrate pseudobinary eutectic was predicted by Zeng and Voigt to have a composition of 97 wt % Zn(NO_3_)_2_·6(H_2_O) and 3 wt % NaNO_3_, with a pseudobinary eutectic temperature of 35.05 °C.^4^

Stable and reversible melting and solidification behavior is required for the use of PCMs as TES media. While there exists variability between the behavior observed in specific salt hydrate systems, salt hydrates in general have exhibited two problematic features: (1) the tendency of incongruent melting to result in phase segregation (and thus a reduction in the observed enthalpy at melting with repeated cycling) and (2) the tendency of many salt hydrates to exhibit nucleation-limited undercooling behavior. An example of incongruent melting is calcium chloride hexahydrate, where stoichiometric crystalline CaCl_2_·6(H_2_O) experiences a peritectic reaction (CaCl_2_·6(H_2_O) → CaCl_2_·4(H_2_O) + brine) prior to reaching the liquid temperature.^22^ Such a reaction results in liquid and solid phases which are compositionally dissimilar, and thus potentially subject to buoyancy-driven phase segregation processes. Nucleation-limited undercooling behavior affects salt hydrates and related eutectics as they can remain in a metastable liquid state by a few to a few tens of degrees below the equilibrium melting temperature, resulting in unpredictable solidification behavior. As an example, small volumes of lithium nitrate trihydrate (<10 μL) can undercool to approximately 40 °C below its melting point upon cooling at 2 °C·min^–1^. Similar volumes of Ca(NO_3_)_2_·4(H_2_O) can be undercooled to a greater value of about 65 °C when cooled at 1 °C·min^–1^.^23,24^ Eutectics presents unique challenges, due to the fact that two crystalline phases are simultaneously solidifying. At equilibrium, the aggregate solidifying composition would be equal to that of the liquid; however, as noted previously, the extent of undercooling may be dissimilar in the different phases solidifying, which could feasibly introduce concerns with phase segregation. The interaction between undercooling and phase segregation in a system that is crystallizing two or more phases is currently poorly understood.^6^

Undercooling in phase change materials introduces stochastic and unpredictable behavior on cooling and reduces the efficiency of a thermal energy storage system. Some classes of organic PCMs, including paraffins, tend to have little to no undercooling.^22,25^ In contrast, inorganic PCMs, including salt hydrates and low-melting point metals and alloys, as well as other organic PCMs, including sugar alcohols, experience high undercooling due to the large interfacial energy between the solid and liquid phases. In both cases, heterogeneous nucleation tends to occur from impurities which introduce heterogeneous nucleation cites with a range of nucleation potencies.^26^ These undercooling tendencies result in varying nucleation rates between the different classes of PCM which can be extrapolated to infer solidification behavior in different volumes and at different cooling rates.^8,27−29^

The lack of nucleation sites during solidification may be addressed in part by adding nonreactive nucleation particles (NPs) with low interfacial energy to the solid phase, which therefore tend to promote nucleation. In the past, strategies to deliberately select NPs have been developed based on either epitaxial lattice match or an isostructural relationship, or having a similar chemical composition to that of the solidifying material.^30−33^ This principle of adding nucleation sites in materials by way of seeding NPs has been developed extensively in metallic systems to enable preferential phase growth in stainless steel or in biological systems to promote the crystallization of protein crystals.^34,35^ An example of a salt hydrate and a NP pairing is the mineral likasite, Cu_3_(NO_3_)(OH)_5_·2(H_2_O), which has a small degree of lattice disregistry for lithium nitrate trihydrate, LiNO_3_·3(H_2_O), resulting in undercooling in small volumes (<10 μL) reduced from 40 °C to as little as 6 °C, at a cooling rate of 10 °C·min^–1^, with less than 1 wt % of the NP present.^23,36^ In eutectics, more than one NP can be added to the system to promote nucleation in the different phases which will solidify, as seen in our previous work with lithium nitrate trihydrate eutectics.^8^ With likasite, a mixture of both likasite and a blend of carbonates (BaCO_3_, CaCO_3_, and SrCO_3_) was added to the LiNO_3_·3(H_2_O)-NaNO_3_ eutectic, where the carbonate mixture aided the nucleation of the NaNO_3_ present and allowed for both LiNO_3_·3(H_2_O) and NaNO_3_ to nucleate at similar degrees of undercooling.

Zinc nitrate hexahydrate, Zn(NO_3_)_2_·6(H_2_O), henceforth referred to as ZNH, is a congruently melting salt hydrate with a previously reported melting temperature of 36.4 °C and a *ΔH*_fus_ of 147 J·g^–1^, and it does not thermally decompose until above 300 °C.^18,37,38^ A eutectic between Zn(NO_3_)_2_·6(H_2_O)and Zn(NO_3_)_2_·4(H_2_O) exists at approximately 34.6 °C, with a Zn(NO_3_)_2_ concentration of 66.2 wt %.^39−41^ ZNH is hygroscopic, resulting in an increase in water concentration to greater than the stoichiometric ZNH concentration if it is handled under ordinary laboratory conditions in most cases. Without any NP present, ZNH can stochastically nucleate at an undercooling greater than 20 °C at times, with a cooling rate of 10 °C·min^–1^ and a volume of approximately 10 μL. Thickeners can be added to salt hydrates and their eutectics to provide shape stabilization upon phase transformations, which is needed in TES media; one identified thickener for ZNH is carboxymethyl cellulose, as the polymer can dissolve in the ZNH.^42^ Zinc oxide, ZnO, and zinc hydroxide, Zn(OH)_2_, were proposed as NPs for ZNH, with their presence in the salt hydrate reducing undercooling to 1–6 °C.^18,32,37,43^ A NP must also be evaluated for its stability over time by way of aging the NP in the salt hydrate, observing if any chemical reactions occur that would degrade the nucleating ability.

In this work, stoichiometric ZNH, as well as three ZNH pseudobinary eutectics, ZNH-NH_4_NO_3_, ZNH-KNO_3_, and ZNH-NaNO_3_, are developed and evaluated to understand the extent and effect of metastability and phase segregation phenomena in salt hydrate eutectic systems. ZNH eutectic compositions and their thermophysical properties are determined as well as their thermal stability upon cycling. The abundant geological mineral talc is investigated as an NP for ZNH and its eutectics and is shown to decrease undercooling to a similar extent in ZNH and all of the investigated eutectics. The stability of these eutectic systems is then evaluated by way of cycling and aging to demonstrate that these ZNH eutectics can withstand hundreds of thermal cycles, while also demonstrating the long-term stability of talc as an NP for ZNH eutectics. Through this study, we advance the understanding of the effects of metastability and phase segregation on salt hydrate eutectics while also developing a new system with potential applications for building thermal energy storage.

## 2 Method

### 2.1 Materials

**Figure 2:**
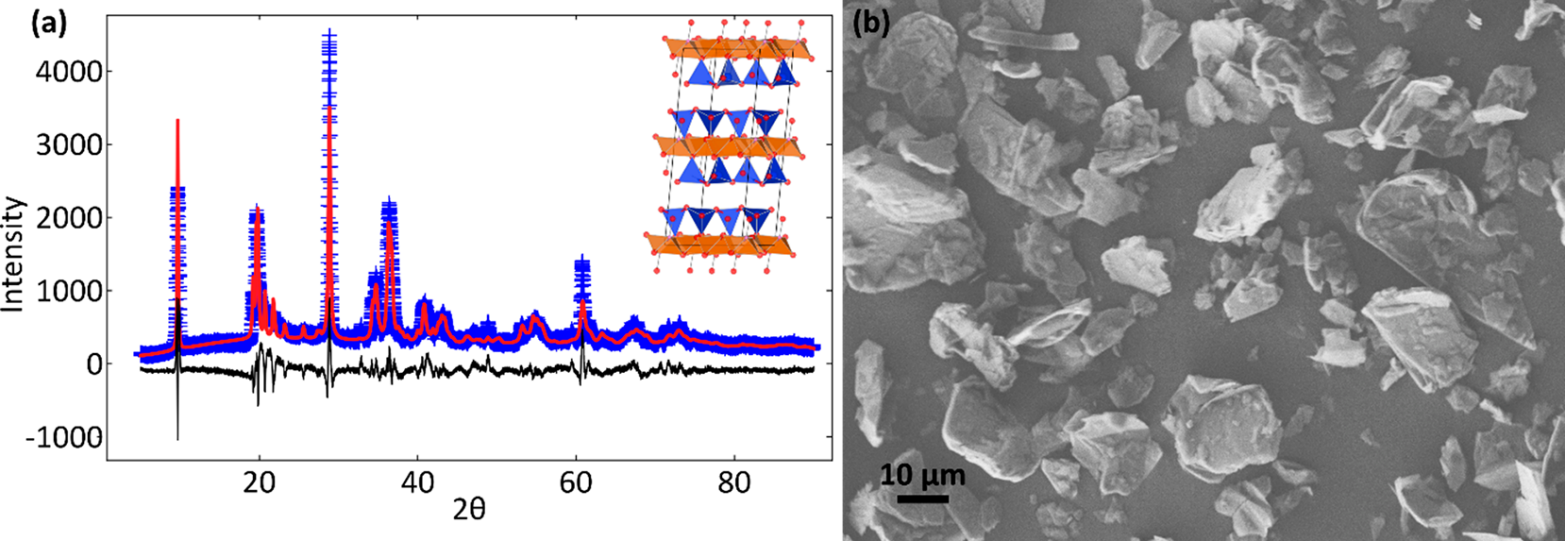
(a) The observed powder diffraction pattern of talc in blue, with the calculated spectra illustrated in red, and a difference curve in black.^49^ A supercell of the resultant crystal structure of talc in the top right corner, showing the layering of tetrahedral-octahedral-tetrahedral sites observed in talc, resulting in a pseudohexagonal in-plane structure. (b) SEM image of talc taken using secondary electrons at 10 keV. The image shows the flaky nature of talc.

Zinc nitrate hexahydrate, Zn(NO_3_)_2_·6(H_2_O) (purum p.a., crystallized, ≥ 99.0%, Sigma-Aldrich) was melted and recrystallized at 33.5 °C for 24 h to promote crystallization of the hexahydrate phase, after which the supernatant was removed and the remaining crystal was used in determining and producing eutectic compositions, thermal conductivity, nucleation and aging studies, and cycling testing. A 99.998% metals basis (Puratronic) ZNH from Alfa Aesar was also used to determine the thermophysical properties of ZNH. Variance in melting behavior was seen between samples in as-received high-purity ZNH where some samples displayed a single melting peak, whereas others exhibited a secondary lower temperature peak which is consistent with the Zn(NO_3_)_2_·6(H_2_O) - Zn(NO_3_)_2_·4(H_2_O) eutectic temperature (SI Figure 1).^18,37^ Recrystallization of the starting as-received material was observed to generally decrease initial variability in water concentration in as-received material, tending to result in water concentrations closer to the desired stoichiometric phase; this approach was consistent with the previously described strategy of purifying LiNO_3_·3(H_2_O) based eutectics.^8^ However, even this recrystallization process did not always result in stoichiometric water concentrations, as in some cases, a small endothermic peak is observed below the melting peak of pure ZNH. This lower-temperature peak tended to be more prevalent at faster scanning rates. Polarized light microscopy supports that this precursor melting peak is associated with incipient melting at the eutectic temperature, and is indicative of excess Zn(NO_3_)_2_ present in the salt hydrate (SI Figure 2). Ammonium nitrate (NH_4_NO_3_; 95% min), and sodium nitrate (NaNO_3_; 99.0% min) were received from Alfa Aesar; potassium nitrate (KNO_3_; Granular/Laboratory grade), was received from Fisher Scientific. Natural talc (Mg_3_Si_4_O_10_(OH)_2_) was obtained from Aldon Corporation; X-ray diffraction confirmed the relative phase purity of the obtained talc (Figure 2). As talc is hygroscopic, it was dried in a vacuum oven at 200 °C for at least 8 h before its addition to salt hydrates.

### 2.2 Materials Characterization

A TA Instruments Q2000 and Setaram Microcalvet DSC were used to measure the melting temperature (*T*_fus_) for stoichiometric ZNH, the eutectic temperature (*T*_eu_) for eutectic compositions, the enthalpy of fusion (Δ*H*_fus_), and the eutectic enthalpy (Δ*H*_eu_). The Q2000 DSC was used for eutectic composition scanning, NP evaluation, and aging studies; the Microcalvet was used in thermophysical property determination and cost density calculations. The Q2000 calorimeter was calibrated with a pure indium standard and pure tin standard (w = 0.9999), using reference values of *ΔH*_fus_ = 28.662 J·g^–1^ for indium and *ΔH*_fus_ = 60.216 J·g^–1^ for tin, and *T*_fus_ = 156.598 °C for indium and *T*_fus_ = 231.9 °C. Predicted relative uncertainties (*u*_r_) for individual measurements at a 95% confidence interval, based on repeated analysis of the aforementioned standards for the Q2000 are *u*_r_(*T*_fus_) = ± 0.001 and *u*_r_(Δ*H*_fus_) = ±0.026 for indium, and *u*_r_(*T*_fus_) = ±0.0006 and *u*_r_(Δ*H*_fus_) = ±0.032 for tin. Deviations between reported sample averages and reference values of the Q2000, , are within ±0.0002 and ±0.04 for *T*_fus_ and Δ*H*_fus_ respectively. The Microcalvet was calibrated with water and naphthalene standards, using reference values of *ΔH*_*fus*_ = 334 J·g^–1^ and *ΔH*_*fus*_ = 147.639 J·g^–1^, and *T*_fus_ = 0 °C for and *T*_fus_ = 80.25 °C for water and for naphthalene, respectively. Predicted relative uncertainties (*u*_r_) for individual measurements at a 95% confidence interval, based on repeated analysis of the water standard for the Microcalvet are *u*_r_(*T*_fus_) = ±0.0009 and *u*_r_(Δ*H*_fus_) = ±0.04. Deviations between reported sample averages and reference values of the Microcalvet, , are within ±0.0001 and ±0.001 for *T*_fus_ and Δ*H*_fus_, respectively.

Q2000 samples were measured in hermetically sealed aluminum pans from TA Instruments, and sealed stainless steel sample holders for the Microcalvet, mitigating changes in composition due to absorption or loss of water vapor to the ambient environment and ranged from 8 to 13 mg in mass. Microcalvet samples, which were used to report final enthalpies and transition temperatures were prepared under dry N_2_ atmospheres. Samples underwent heating and cooling cycles in a nitrogen environment, with a continuous nitrogen flow of 50 mL·min^–1^, and at a temperature ramp rate of 10 °C·min^–1^ during preliminary analysis in the Q2000, and 1 °C·min^–1^ for the Microcalvet. The former, faster scan rate allowed for quicker scans to rapidly converge on eutectic compositions, whereas the slower rate was more suited for final analysis completed in the Microcalvet system. Following ASTM E794–06, *T*_fus_ and *T*_eu_ values are defined as the onset of the melting curve, defined as the intersection between the baseline and the tangent of the melting curve with the steepest slope.^44^ The onset temperature of endothermic melting peaks was adopted as the eutectic point as the eutectic is an invariant point. As with other invariant points, the peak temperature is sensitive to heating rate (SI Figure 3).^45^ ASTM E793–06 was followed to determine *ΔH*_*fus*_ through the integration of the melting peak in samples tested.^46^ Undercooling, Δ*T*, was reported as the difference between *T*_eu_ and crystallization temperature *T*_crys_, with *T*_crys_ being defined as the onset of the abrupt crystallization exothermic peak.

Liquid density calculations were taken at room temperature (approximately 21 °C) by measuring the mass of a calibrated 10 μL droplet using an Eppendorf micropipette. The micropipette volume was calibrated with ultrapure water (ρ_L_ = 0.999 g·cm^–3^), resulting in an estimated density uncertainty of *u* = ±0.01 g·cm^–3^.^47^ Crystallographic densities are calculated from crystal structures resolved by X-ray diffraction techniques, assuming negligible porosity.

Liquid thermal conductivity measurements were taken using a hot-wire (Thermtest THW-L2) following ASTM D7896–19 in conjunction with a dry bath (Torrey Pines Scientific Echotherm Chilling/Heating Dry Bath) to control the temperature of the sample while measurements were taken.^48^ Samples were inserted inside an aluminum cylinder with spacers that enabled sample volumes as small as 15 mL. As hot-wire systems are limited to measuring liquid samples, the thermal conductivity was measured only in the liquid phase. A Parylene C coating with an approximate thickness of 4 μm was coated on the sensor wire in the hot wire probe to prevent electrical conductivity or any other electrochemical reactions through liquids with ionic conductivity inside the sample holder. Deionized water (DIW) was repeatedly measured over the same temperature range as investigated in this study, resulting in an estimated uncertainty of *u* = ±0.03 W·m^–1^·K^–1^ for the hot-wire system.

X-ray diffraction (XRD), scanning electron microscopy (SEM), and energy-dispersive spectroscopy (EDS) were used to characterize the talc powder and compare them to prior characterizations of talc reported in literature.^49^ An X-ray powder diffractometer (Bruker D8 Bragg–Brentano) was used for XRD using a Cu Kα beam; FE-SEM and EDS (JEOL JSM-7500F) were used to confirm the structure of talc particles using an accelerating voltage of 10 keV with secondary electrons. The resultant XRD spectra were refined using GSAS-II which then a resultant crystal structure was generated using VESTA (Figure 2a), accounting for all the major diffraction peaks, with resultant lattice parameters reported in Table 1.^49^ EDS confirmed the majority presence of magnesium, oxygen, and silicon within the material; however, minor quantities of aluminum are also observed. The SEM images reveal flaky particles, consistent with the layered crystal structure of the phyllosilicate (Figure 2b). XRD was also conducted to compare both the dried and as-received talc to show that there was no changes in structure after it was dried at 200 °C (SI Figure 4) which is consistent with prior studies which indicated a stable structure in talc to at least 400 °C.^50^

**Table 1:** Lattice Parameters of Talc, Resolved by X-ray Diffraction

	Lattice Parameters												
Space Group	*a* (Å)	2σ	*b* (Å)	2σ	*c* (Å)	2σ	α (deg)	2σ	β (deg)	2σ	γ (deg)	2σ	ref.
*P*1̅	5.291	0.004	9.162	0.003	9.49	0.01	90.58	0.06	99.672	0.03	90.06	0.02	This work
*P*1̅	5.29	0.03	9.17	0.0	9.46	0.05	90.46	0.05	98.68	0.05	90.09	0.05	(49)

### 2.3 Thermal Cycling and Isothermal Nucleation

Cycling and isothermal transformation were completed in a stainless steel apparatus composed of 10.2 cm long (8 mm inner diameter) 304 stainless steel tubes and compression fittings. A stainless steel shielded, ungrounded 15.2 cm long K-Type thermocouple (Omega, *u* = ±0.01 °C) was inserted axially down the center of each tube, and the temperature was recorded using a data acquisition board (DAQ; Omega, OMB-DAQ-2408) at a frequency of 0.5 Hz. Teflon spacers were used approximately 1 cm from the tip to prevent the displacement of the thermocouple from the central axis. Each stainless steel cell was filled with approximately 3 mL of liquid salt hydrate and 2 wt % talc when applicable under a dry N_2_ environment and then was hermetically sealed using compression fittings. Cycling and isothermal experiments were completed by placing these tubes, henceforth known as cells, in a programmable recirculating water bath (PolyScience Refrigerated Circulator AP07R-40-A11B) filled with a polyethylene glycol/water mixture. An identical thermocouple was placed into the water bath to measure the water temperature during each experiment. Cycling programs were completed with temperature ramp rates of approximately 1 °C·min^–1^. In cases where the low-temperature end was below 0 °C, a 5 min hold was appended to the cooling portion to ensure the water bath was able to reach the desired minimum temperature. Isothermal testing began with samples thermally equilibrated 15 to 25 °C above their eutectic melting point for 15 min before being placed in the water bath at the chosen isothermal temperature some degree below the melt temperature. The time of the onset of crystallization was observed by monitoring thermocouple readings for abrupt temperature increases associated with exothermic crystallization. To compensate for the finite time to cool down to the desired isothermal temperature, the beginning time of the isothermal hold, *t*_iso_, was defined as the time at which the cell thermocouple recorded 0.5 °C above the target isothermal hold temperature.

## 3 Results & Discussion

### 3.1 Thermodynamic Equilibria

**Figure 3:**
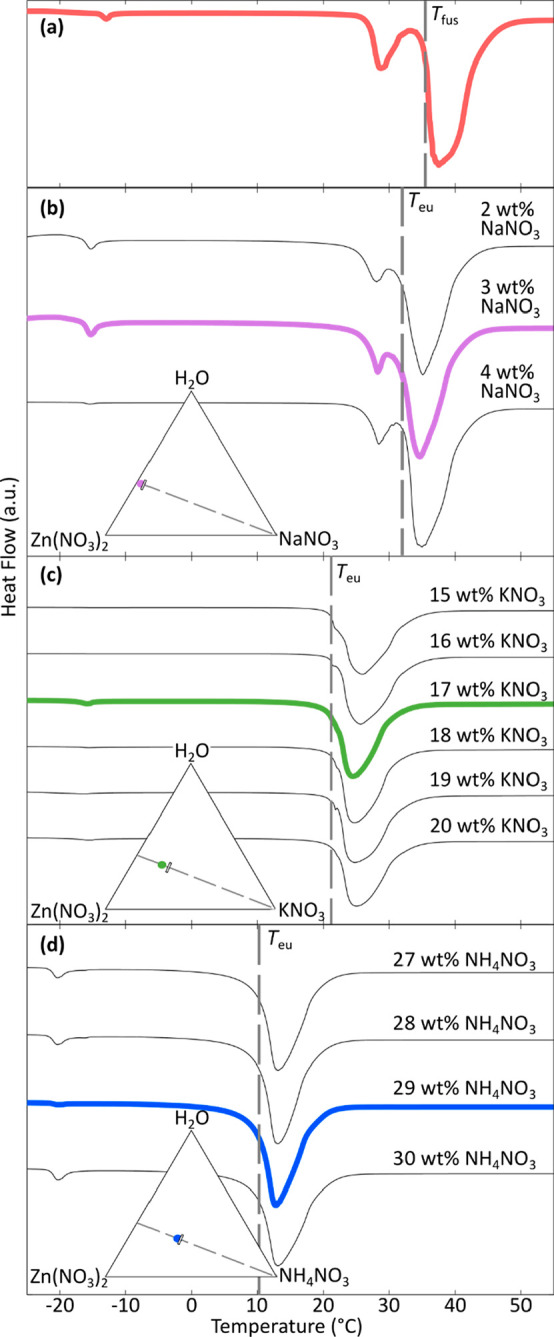
DSC curve of ZNH (a) as well as DSC curves for composition arrays of tested pseudobinary ZNH eutectics, ZNH-NaNO_3_ (b, purple), ZNH-KNO_3_ (c, green), and ZNH-NH_4_NO_3_ (d, blue), as measured at heating and cooling rates of 10 °C·min^–1^ to determine the eutectic composition to within 1 wt % (highlighted in bold and in color). Dashed lines refer to the reported *T*_fus_ or *T*_eu_ collected from the Q2000 DSC. Eutectic compositions were selected for having a narrow peak width, indicative of minima in the liquidus temperature (associated with the eutectic point). Mixtures with higher concentrations of anhydrous nitrate are above the solubility limit at room temperature. A ternary phase diagram is shown on each plot with a dot indicative of eutectic composition, and a tick mark indicative of solublity limit.

**Figure 4:**
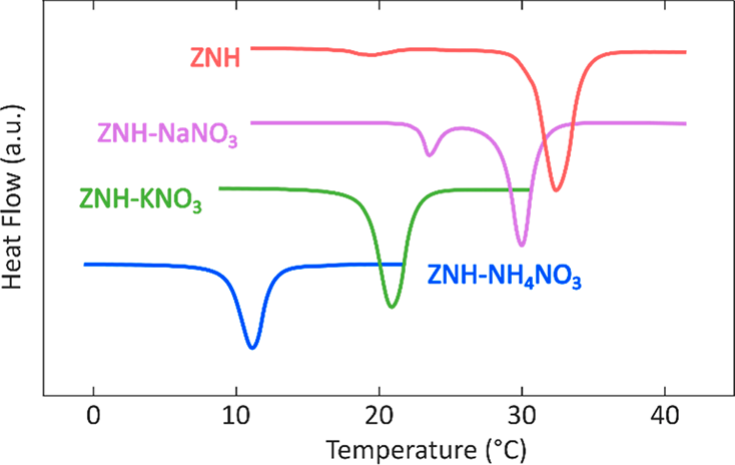
Resultant DSC curves of ZNH and related eutectics taken at 1 °C·min^–1^ on Microcalvet DSC.

Melting characteristics based on the average of 1 to 3 quantities of independently prepared samples of ZNH and the ZNH-NH_4_NO_3_, ZNH-KNO_3_, and ZNH-NaNO_3_ eutectics were evaluated using DSC at a ramp rate of 10 °C·min^–1^ to find eutectic compositions and the thermophysical properties collected at 1 °C·min^–1^ are reported in Table 2 respectively. As the concentration of anhydrous nitrate increases in a ZNH eutectic, the *T*_eu_ and *ΔH*_eu_ of the eutectic decrease with concentration compared to stoichiometric ZNH. The uncertainty associated with variance between individual batches is larger than the instrument uncertainty, indicating that it is derived from the preparation of batches of eutectics with slightly different compositions. Both liquid- and solid-state densities of ZNH and related eutectics are reported in Table 3, which are used to calculate volumetric energy densities (Table 4). DSC scans, at a ramp rate of 10 °C·min^–1^, were used to evaluate different compositions to identify pseudobinary eutectic compositions, where the composition with the lowest difference between the onset temperature and the temperature at the peak of the curve (Figure 3). Final thermodynamic properties were attained from high-purity batches of eutectics and analyzed using a higher accuracy microcalorimeter, based on a Calvet sensor, at a ramp rate of 1 °C·min^–1^ (Figure 4). These results supported the appropriate identification of eutectic compositions by illustrating sharp, single-maxima peaks, indicating an invariant melting eutectic point at that composition.

**Table 2:** Thermophysical Properties of ZNH and Related Eutectics at 1 °C·min^–1^

	*w*_salt_	*N*	*T*_fus_, *T*_eu_^a^			*ΔH*_fus_, *ΔH*_eu_^a^			*ΔH*_fus_, *ΔH*_eu_ (Volumetric)				*ΔS*_f_, *ΔS*_eu_		ref
									Solid		Liquid				
			°C			J·g^–1^			J·cm^–3^		J·cm^–3^		J·kg^–1^ K^–1^		
			Average	2σ^b^	δ (%)^c^	Average	2σ^b^	δ (%)^c^	Average	2σ^b^	Average	2σ^b^	Average	2σ^b^	
Zn(NO_3_)_2_·6(H_2_O) (99% Purity)	0.637	2	34.4	1.1	–0.06	148	0.4	0.01	293	1	260	12	465	1.4	(18,37)
Zn(NO_3_)_2_·6(H_2_O) (99.995% Purity)		2	34.9	0.1	–0.04	152	2	0.03	293	4	261	12	481	6.4	(18,37)
Zn(NO_3_)_2_·6(H_2_O) [0.97]/NaNO_3_ [0.03]	0.647	2	32.67	0.08	-	151	6	-	300	12	272	12	493	19	
Zn(NO_3_)_2_·6(H_2_O) [0.83]/KNO_3_ [0.17]	0.698	1	22.09	-	-	140	-	-	280	1	257	8	474	-	
Zn(NO_3_)_2_·6(H_2_O) [0.71]/NH_4_NO_3_ [0.29]	0.742	3	11.23	0.07	-	137	1	-	260	2	224	8	481	4	

a Predicted relative uncertainties (*u*_r_) for individual measurements at a 95% confidence interval are *u*_r_(*T*_fus_) = ±0.0009 and *u*_r_(Δ*H*_fus_) = ±0.04.

b Reported 2σ uncertainties are based on repeated analysis using independent samples.

c Deviation from previously reported value, as calculated by δ = (*X* – *X*_ref_)/*X*_ref_.

**Table 3:** Measured Liquid Densities and Calculated Crystallographic Solid Densities^a^ of ZNH and Related Eutectics

	Liquid Density		Solid Density		ref.
	g·cm^–3^		g·cm^–3^		
	μ	2σ	μ	2σ	
Zn(NO_3_)_2_·6(H_2_O)	1.76	0.08	1.979	0.001	(51)
Zn(NO_3_)_2_·6(H_2_O) [0.97]/NaNO_3_ [0.03]	1.80	0.04	1.986	0.001	(52)
Zn(NO_3_)_2_·6(H_2_O) [0.83]/KNO_3_ [0.17]	1.83	0.06	1.999	0.001	(53)
Zn(NO_3_)_2_·6(H_2_O) [0.71]/NH_4_NO_3_ [0.29]	1.64	0.06	1.889	0.01	(54)

a From reported references.

**Table 4:** Cost Densities of ZNH and Related Eutectics

	Component Cost			
	ZNH	XNO_3_	Total Cost	Cost-Energy Density
	$·kg^–1^	$·kg^–1^	$·kg^–1^	$·kWh^–1^
Zn(NO_3_)_2_·6(H_2_O)	0.63	n/a	0.63	15.32
Zn(NO_3_)_2_·6(H_2_O) [0.97]/NaNO_3_ [0.03]	0.63	0.24	0.62	14.74
Zn(NO_3_)_2_·6(H_2_O) [0.83]/KNO_3_ [0.17]	0.63	0.20	0.56	14.32
Zn(NO_3_)_2_·6(H_2_O) [0.71]/NH_4_NO_3_ [0.29]	0.63	0.24	0.52	13.58

The Δ*H*_fus_ for the ZNH-NaNO_3_ eutectic has thermophysical properties comparable to that of ZNH but with a eutectic melting point (*T*_eu_ = 32.7 ± 0.3 °C) approximately two degrees below the melting point of ZNH (*T*_fus_ = 34.9 °C). With there being such a low concentration of NaNO_3_ present in this eutectic (3 wt %), its resultant *T*_eu_ is close to the *T*_m_ of ZNH. This system also displayed a small, endothermic peak in all compositions tested. The other two eutectics evaluated had much lower *T*_eu_ values due to higher concentrations of anhydrous nitrate present to suppress the eutectic temperature. Previously, Schmit et al. reported the composition of the ZNH-NH_4_NO_3_ eutectic to be 75% ZNH and 25% NH_4_NO_3_, with a *T*_eu_ of 12.4 ± 0.7 °C, *ΔH*_eu_ of 135 ± 7 J·g^–1^, and density of 1.76 ± (6 × 10^–5^) g·cm^–3^, where *ΔH*_eu_ aligns with the value reported here but the composition differs.^5^ In this study, we saw a lower eutectic point (*T*_eu_ = 11.2 ± 0.3 °C) than that previously reported, and that may be due to the slightly higher concentration of NH_4_NO_3_.

The solubility limits of each nitrate mixture were determined by observing precipitate formation in certain compositions while the mixture was held at a temperature above the system’s eutectic temperature (50 °C) for extended periods. The ZNH-NH_4_NO_3_ system displayed solubility limits at 69 wt % ZNH, ZNH-KNO_3_ at 79 wt % ZNH, and ZNH-NaNO_3_ at 95 wt % ZNH as they were held at 50 °C.

Cost-energy densities are reported in Table 4 based on bulk costs from an industrial supplier. For this data, the costs of salt hydrates and anhydrous salts are evaluated from a single retailer (Alibaba.com), using an average of the three lowest provided costs that were able to supply industrial-grade material in bulk (>10^3^ kg). The eutectics have a lower cost-energy density compared to stoichiometric ZNH but the ZNH-NaNO_3_ and ZNH-KNO_3_ have higher volumetric energy than ZNH due to the eutectics having comparable *ΔH*_fus_ of the system and the anhydrous nitrates decreasing overall cost. The ZNH-NH_4_NO_3_ eutectic has the lowest cost-energy density and the ZNH-NaNO_3_ eutectic has the highest volumetric energy density of the three eutectics presented.

### 3.2 Thermal Conductivity of ZNH Eutectics

**Figure 5:**
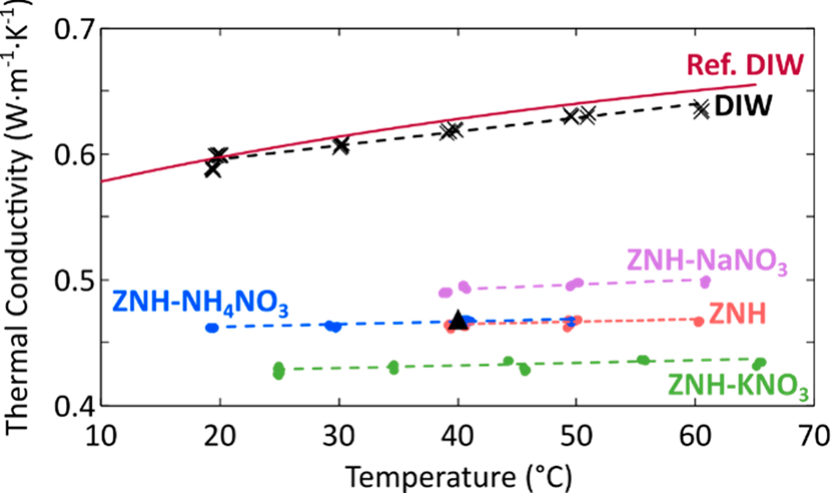
Thermal conductivity of liquid ZNH and related eutectics, compared to water, measured on both heating and cooling. Deionized water (DIW) is provided for comparison as well as reference values from NIST.^55^ The black triangle at 40 °C is a previously reported thermal conductivity for ZNH from Lane.^3^

Temperature-dependent thermal conductivities of liquid ZNH and related eutectics were determined using the hot-wire method following ASTM D7896–19 (Figure 5).^48^ The temperature-dependence of thermal conductivity is approximately linear over this range and is fit to the expression: *k*(*T*) = *k*_0_(1 + *α*_*k*_(*T* – *T*_0_)) (Table 5). All data was measured on heating and cooling to demonstrate repeatable behavior. All the eutectics displayed high thermal conductivity values greater than 0.4 W·m^–1^·K^–1^, but substantially lower thermal conductivity than DI water. The effect of adding different anhydrous nitrates (NaNO_3_, KNO_3_, NH_4_NO_3_) depended on the nitrate species added and was independent of the concentration of that secondary component; thermal conductivity was either increased or decreased relative to that of ZNH, based on the nature of the anhydrous nitrate added. The Zn(NO_3_)_2_·6(H_2_O)-NaNO_3_ system displayed the highest thermal conductivity of the ZNH systems.

**Table 5:** Temperature Coefficient of Thermal Conductivity in ZNH-Based Eutectics

	*k*_0_^a^	*α*_k_ (W·m^–1^·K^–2^)	2σ^b^	*R*^2^ Value
Zn(NO_3_)_2_·6(H_2_O)	0.46	1.9 × 10^–4^	5.1 × 10^–5^	0.46
Zn(NO_3_)_2_·6(H_2_O)-NaNO_3_	0.49	3.5 × 10^–4^	6.5 × 10^–5^	0.63
Zn(NO_3_)_2_·6(H_2_O)-KNO_3_	0.43	2.1 × 10^–4^	3.6 × 10^–5^	0.54
Zn(NO_3_)_2_·6(H_2_O)-NH_4_NO_3_	0.47	2.3 × 10^–4^	2.2 × 10^–5^	0.81

a Reported at *T*_0_ = 40 °C.

b 2σ of the slope of fitted trendline

### 3.3 Nucleation in Zn(NO_3_)_2_·6(H_2_O) and Related Eutectics

#### 3.3.1 Evaluation of Different Nucleation Particles

**Figure 6:**
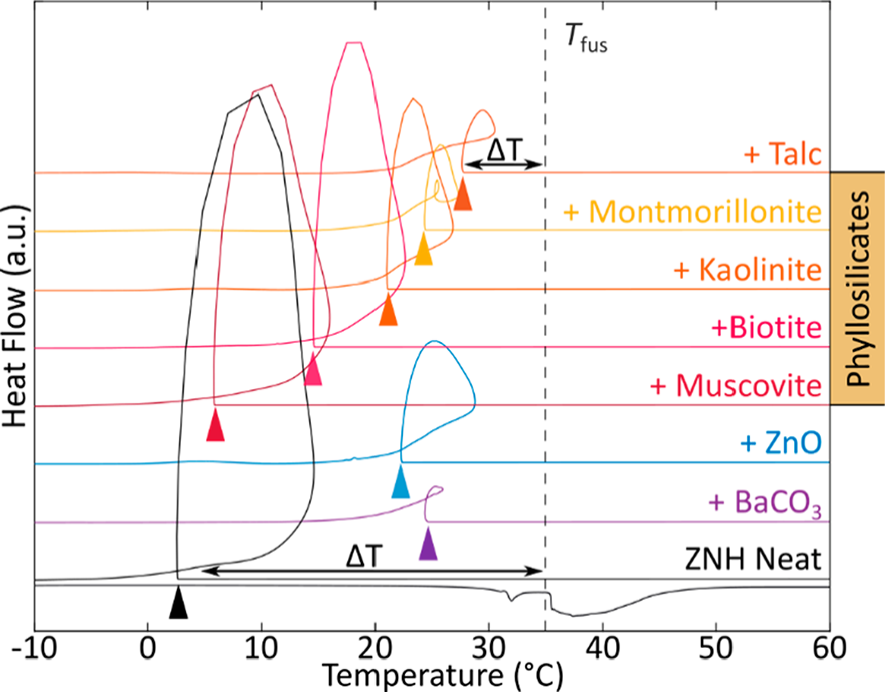
Various NPs were tested alongside neat ZNH to determine the effect on undercooling. 1 wt % quantities of NP were added to a DSC pan with ZNH.

Large degrees of undercooling limit the use of salt hydrates, including ZNH. To combat this effect, secondary solid nucleation particles (NPs) are often added to promote nucleation and thereby decrease undercooling (Figure 6).^56^ Previously, it was reported that zinc oxide, ZnO, is an effective NP for ZNH, reducing the degree of undercooling to as low as 3 °C with large concentrations (7 wt %) of ZnO present.^38^ The large concentration present in this study suggested that it is not a particularly active surface for nucleation. Thus, various potential NPs, including phyllosilicates, carbonates, and the previously mentioned ZnO, were evaluated to find more active NPs. These tests were completed using DSC, with small volumes (<10 μL) and relatively fast ramp rates (10 °C·min^–1^), with 1 wt % quantity of NP included. In addition to talc, other phyllosilicates (layered silicates, including clays and micas) that were tested included biotite, kaolinite, montmorillonite, and muscovite. Kaolinite had the highest undercooling with approximately 26.6 °C of undercooling, and talc had the least with approximately 8.6 °C of undercooling, further confirming the efficacy of talc as a nucleation particle especially considering the lower mass fraction required compared to ZnO. Talc’s high nucleating activity was surprising as it has no apparent crystallographic relationship with ZNH. While comparisons across nucleation experiments that are completed differently are difficult to assess, the observation that talc demonstrated lower undercooling than ZnO under identical conditions, suggested a higher degree of nucleation activity associated with talc surfaces than had previously been observed with ZnO. Additionally, our studies showed that the presence of talc had no measurable effect on the *T*_fus_ or *ΔH*_fus_ on ZNH at 1 to 2 wt % concentrations.

#### 3.3.2 Isothermal Nucleation Kinetics of Zinc Nitrate Hexahydrate Systems

**Figure 7:**
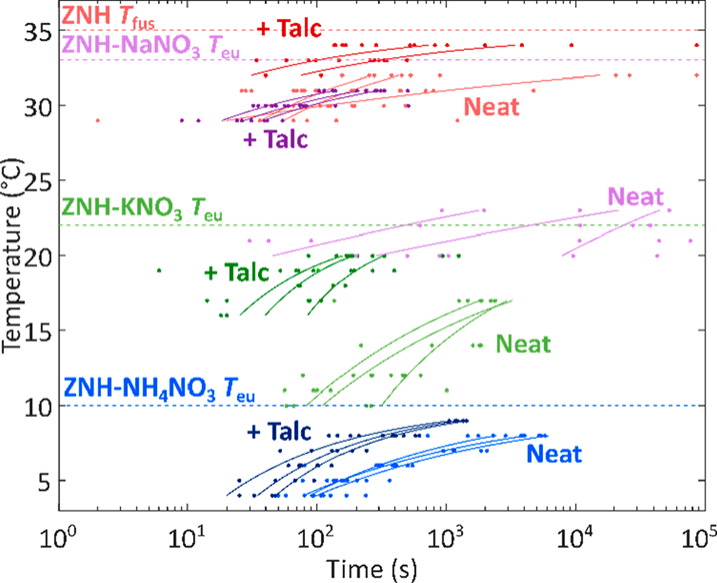
Isothermal crystallization times at various temperatures for ZNH (red) and eutectics containing NaNO_3_ (purple), KNO_3_ (green), and NH_4_NO_3_ (blue) in the presence and absence of the talc nucleation particles. Darker shades of the two colors presented at a temperature represent samples that contain 2 wt % of talc. Curves represent isothermal nucleation times for 25, 50, and 75% probability of nucleation, calculated as described in the text.

Cells were held in an isothermal environment for up to 24 h to derive isothermal nucleation rates for systems at different temperatures in the presence and absence of 2 wt % of talc NPs (Figure 7). Characteristic times (τ_P_) associated with the 25th, 50th, and 75th percentiles of times compiled from repeated isothermal testing and were used to find the characteristic nucleation rates (1/τ_P_) at each percentile. From classical nucleation theory, the nucleation rate, *β*_P_, at a degree of undercooling, *ΔT*, follows the general expression:^57,58^
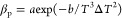
However, in cases where the single most potent catalyst is responsible for nucleation, extreme value statistics may be serve as a more accurate description of the temperature-dependent nucleation process.^26,59^ Here, we adopted a simplified power law relationship, appropriate for investigating nucleation over small degrees of undercooling:^27,60^
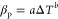
where *β*_P_ is in units of cm^–3^·s^–1^, and the degree of undercooling, *ΔT*, is given in units of K. Experimental parameters, *a* and *b*, were found from the isothermal data collected for ZNH and related eutectic at the 25th, 50th, and 75th percentiles (Table 6). The power law relationship is a phenomenological expression, and has been utilized in several previous studies to model nucleation.^27,60,61^ In this study, the main limitations are due to sample-to-sample variation, likely caused by minor differences in internal defect populations and the stochastic nature of the nucleation phenomenon. Thus, the phenomenological model is sufficient to describe the general temperature dependence of the nucleation rate. Cumulative distribution functions and plots used to find nucleation rates can be found in SI Figures 5–12. Two surprising features arose from the experimentally determined nucleation rates: (1) Intrinsic nucleation rates of pure eutectics varied dramatically, despite relatively small differences in composition. For example, undercooling in the ZNH-NaNO_3_ system was dramatically larger than in pure ZNH, despite the fact that the NaNO_3_ component was relatively minor. (2) Despite these differences, the existence of talc NPs resulted in relatively uniform undercooling in each case. This suggests that the efficacy of talc as an NP is dominated by its role in nucleation of ZNH, rather than the other secondary solid phase present in the eutectic systems. This further suggests that at least in some cases the strategy of including a single NP developed for the major component of the eutectic system is sufficient. This is different from the Li(NO_3_)·3(H_2_O) system, which demonstrated measurable signals for separate nucleation of two different solid phases, and which benefited from the presence of multiple NPs targeting each nucleating phase.^8^

**Table 6:** Experimental Parameters for Nucleation Rates of ZNH and Related Eutectics

Sample			*a*	*b*
Zn(NO_3_)_2_·6(H_2_O)	Neat	β_0.25_	1.43 × 10^–3^	3.779
		β_0.50_	1.05 × 10^–3^	4.055
		β_0.75_	2.37 × 10^–10^	8.786
	+ Talc	β_0.50_	1.34 × 10^–3^	2.864
		β_0.75_	3.39 × 10^–4^	3.532
Zn(NO_3_)_2_·6(H_2_O)-NH_4_NO_3_	Neat	β_0.25_	9.82 × 10^–4^	4.286
		β_0.50_	2.45 × 10^–4^	4.706
		β_0.75_	1.76 × 10^–4^	4.920
	+ Talc	β_0.25_	5.78 × 10^–3^	3.040
		β_0.50_	9.42 × 10^–3^	3.115
		β_0.75_	1.21 × 10^–2^	3.170
Zn(NO_3_)_2_·6(H_2_O)-KNO_3_	Neat	β_0.25_	2.95 × 10^–4^	5.727
		β_0.50_	1.36 × 10^–4^	6.211
		β_0.75_	2.78 × 10^–3^	5.253
	+ Talc	β_0.25_	2.12 × 10^–2^	2.702
		β_0.50_	3.76 × 10^–2^	2.707
		β_0.75_	5.90 × 10^–2^	2.883
Zn(NO_3_)_2_·6(H_2_O)-NaNO_3_	Neat	β_0.25_	2.86 × 10^–9^	11.514
		β_0.50_	1.11 × 10^–7^	10.787
		β_0.75_	5.18 × 10^–4^	7.759
	+ Talc	β_0.25_	1.51 × 10^–2^	2.144
		β_0.50_	1.36 × 10^–2^	2.338
		β_0.75_	1.46 × 10^–2^	2.473

#### 3.3.3 Stability of Nucleation Particles

**Figure 8:**
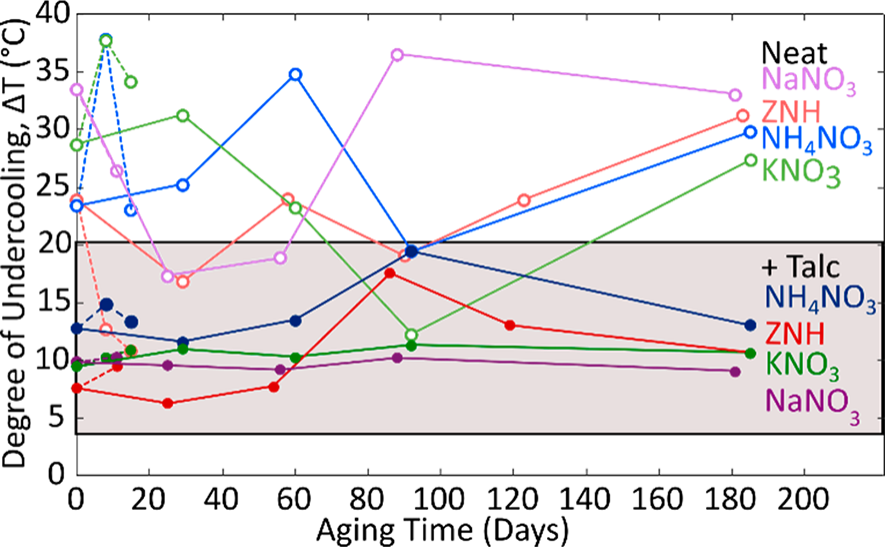
Samples were aged for short periods and periodically sampled in hermetically sealed DSC pans initially (dashed line), and were then extracted from vials and measured (solid line). Neat systems (light shades) were compared with talc-inclusive systems (dark shades).

Heterogeneous nucleation is understood as a process that occurs at the interface between a liquid and some defects (e.g., a solid NP). Thus, dissolution, a chemical reaction, or any other time-dependent process that changes the structure of that solid interface could affect the ability of an NP to nucleate solids in the liquid phase consistently with aging. To assess the stability of talc NPs, samples were tested periodically as they were aged at 50 °C for approximately six months (Figure 8). Two sample sets were tested: one of neat PCM and one combining the PCMs with 2 wt % of talc. Hermetically sealed DSC pans were aged for a short period (15 days) before failing at the compressed interface. Borosilicate glass vials containing samples were aged for >180 days, during which time they were periodically sampled, assuring that some NP was contained in each sampling, which still resulted in minor variability in the concentration of NP contained within each DSC pan during testing.

Over the aging period of six months, the samples were measured using DSC, and their degree of undercooling was determined, where undercooling was measured in a small volume (10 μL) and at relatively rapid cooling rates (10 °C·min^–1^). Samples containing talc had consistent and low degrees of undercooling which remained relatively stable despite prolonged aging. In contrast, neat samples displayed more stochastic behavior and higher degrees of undercooling. The consistent degrees of undercooling found in samples containing talc indicate that talc does not degrade in these eutectics over a long period and retains its potency as a site for heterogeneous nucleation.

### 3.4 Thermal Stability of ZNH and Related Eutectics

**Figure 9:**
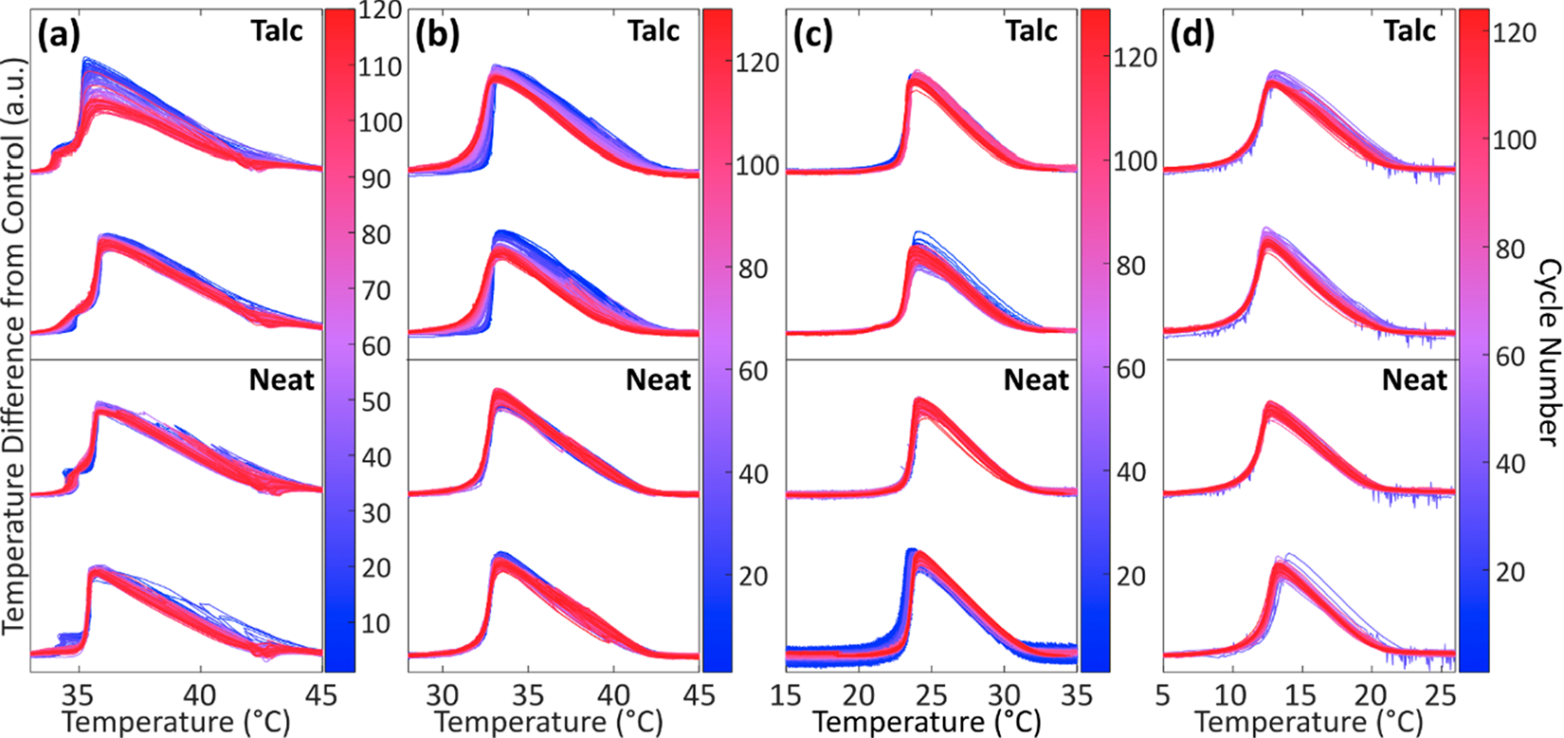
Calculated difference between the cell thermocouple reading and the control thermocouple in an unhoused cell are plotted against temperature for (a) ZNH, (b) ZNH-NaNO_3_, (c) ZNH-KNO_3_, and (d) ZNH-NH_4_NO_3_. The top two curves of each plot are talc-inclusive samples, and the bottom two are neat samples.

**Figure 10:**
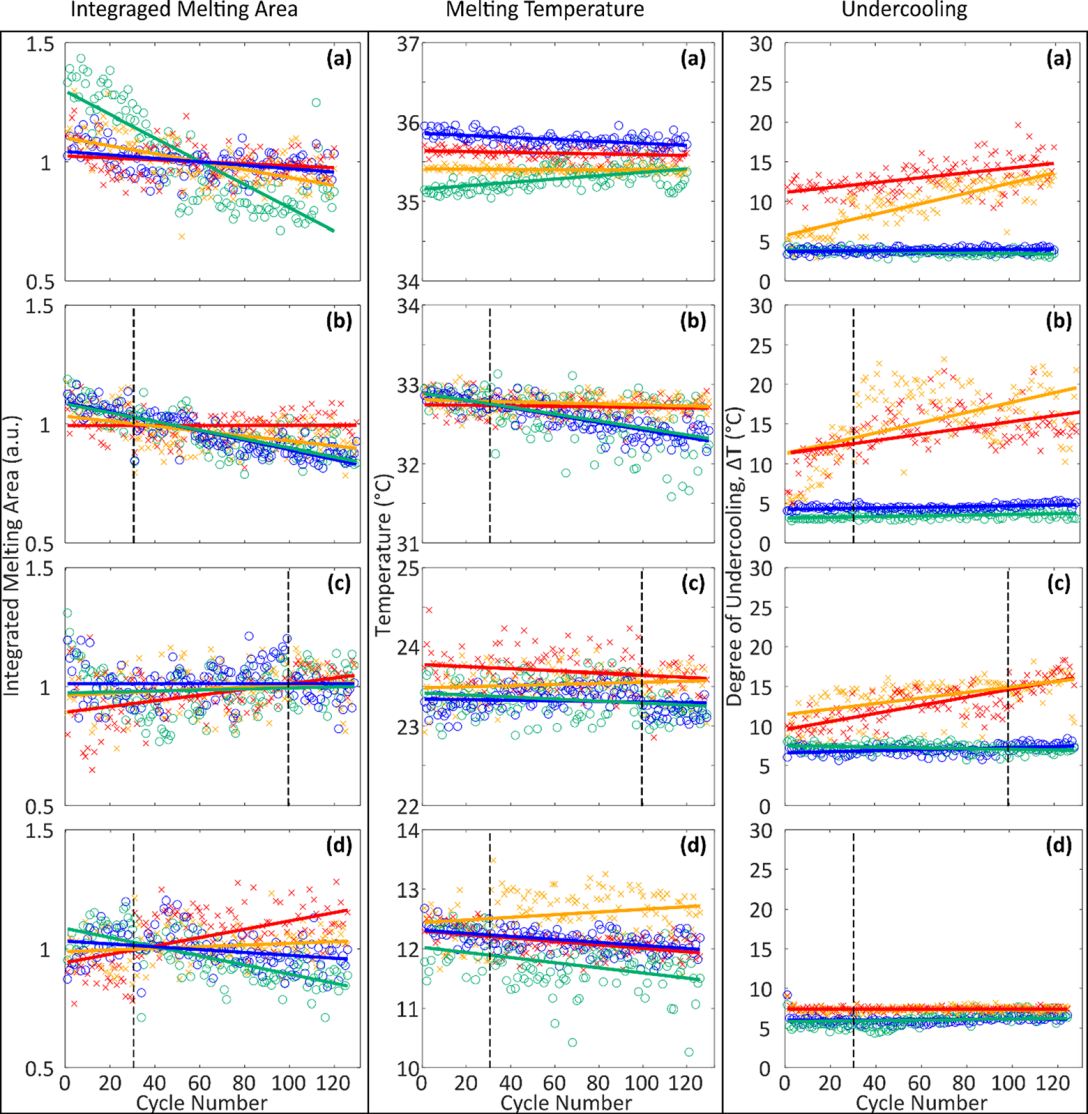
Three sets of graphs for: (left) normalized integrated area of the melting curves, (middle) melting temperature, and (right) degrees of undercooling for (a) ZNH, (b) ZNH-NaNO_3_, (c) ZNH-KNO_3_, and (d) ZNH-NH_4_NO_3_, over many cycles. Red and orange points indicate neat samples A and B respectively for each system, and blue and green indicate talc samples A and B for each system. The vertical line indicates a pause between sets of cycles in all systems but ZNH where it did not have one.

Two cells of each salt hydrate system containing 2 wt % of talc NPs, and two cells lacking NPs were thermally cycled for >120 cycles at approximately 1 °C·min^–1^ from 25 °C above and below their respective melting temperature to observe the complete melting and solidification process, ensuring a equivalent temperature range between all systems. This experiment uses relevant volumes (3 to 5 mL) and serves to detect the signature of phase segregation by consideration of the shape-normalized area and onset temperature of the melting curve, defined as the temperature difference between a specific cell and the surrounding water bath (Figure 9). The integration of the melting curve area, melting temperature, and undercooling of each cycle for each system is compared with each other in Figure 10. These temperature difference curves are similar to differential scanning calorimetry; however, because the volume of the cell is relatively large, the internal temperature deviates significantly from the temperature of the surrounding fluid during melting and solidification. Undercooling values were also recorded for each cycle by subtracting the undercooling temperature from the melting temperature to determine Δ*T*. To further assess the impact of thermal history, in materials with melting temperatures near room temperature, a discrete pause (for a period of a few days) was added, during which time the materials maintained a steady room temperature (indicated by a vertical line on Figure 10). The slopes from the fitted linear trendlines with uncertainties are shown in SI Table 2.

#### 3.4.1 ZNH

The congruently melting ZNH displayed stable melting behavior in most cases, with one notable exception, in which case the melting curve decreased in area systematically over the course of cycling. This may have indicated a minor leak in that cell. but did exhibit altered melting behavior throughout cycles in both the neat and talc cases. This system was cycled for 120 cycles without an extended pause between cycles. The addition of talc to a cell resulted in negligible changes in cycling stability. However, the presence of talc promoted consistently low degrees of undercooling, while neat cells had an increase in undercooling over cycles potentially due to impurities in the ZNH dissolving over cycles.

#### 3.4.2 ZNH-NaNO_3_

Talc-containing ZNH-NaNO_3_ samples illustrated a softening of the melting curve, indicated by a lower temperature and less abrupt onset of melting as well as a melting difference curve that was spread over a broader temperature range. This change progressed slowly but steadily over the course of cycling indicating a slow reaction occurring within the sample cells. These signatures are all indicative of minor compositional changes in the eutectic composition and a shift away from eutectic melting behavior that occurs at a single invariant temperature. The observation that this form of degradation occurs in cells that contain talc but not in neat cells is a signal that there may be a potential reaction between talc and NaNO_3_, including a potential ion-exchange reaction. The undercooling behavior of this eutectic showed that the talc cells had low and consistent degrees of undercooling, and the neat degree of undercooling increased over cycles. Despite the benefit that talc brings to this system’s degree of undercooling, the potential reaction degradation of thermal properties suggests that talc may not be an appropriate NP for use in this system. Further evaluation is ongoing.

#### 3.4.3 ZNH-KNO_3_

The ZNH-KNO_3_ system has a near-room-temperature eutectic temperature of 22 °C, making it especially susceptible to segregation when it is maintained at room temperature in the two-phase regime. This system was initially cycled 90 times, aged for 1 week at 50 °C due to concerns from phase segregation already evident at initial cycles, and then ran for another 30 cycles. Despite this concern, across all cycles, the ZNH-KNO_3_ eutectic showed stability in both talc-containing and neat cells. Like the ZNH-NaNO_3_ eutectic, the talc cells had consistently low undercooling, and the neat cells demonstrated a larger degree of undercooling. The existence of a prolonged time held at room temperature (approximately 21 °C) did not significantly impact the melting behavior.

#### 3.4.4 ZNH-NH_4_NO_3_

ZNH-NH_4_NO_3_ cells were cycled for 30 cycles and then for another 96 cycles, being stored at room temperature between the two sets of cycles. Cells with talc show a minor decrease in the area of the melting curve with cycling, but not to a dramatic degree. Undercooling in the talc cells was lower than the neat, but not by a lesser margin than in other salt hydrate systems. Changes in melting temperature across cycles were minimal for this system. The findings from cycling this system show that talc made less of an impact than it did on other eutectics.

## 4 Conclusions

Here, we investigated a family of pseudobinary eutectics based on zinc nitrate hexahydrate (ZNH), in combination with different anhydrous nitrate salts. ZNH and related eutectics have been identified as a favorable family of PCMs for use in building thermal energy storage applications, as they transform near room temperature (*T*_eu_ = 10 to 35 °C) with low cost-energy densities ≤ $15/kWh while having enthalpies >130 J·g^–1^ and >60 J·cm^–3^. This combination of attributes is ideal for low-cost thermal energy storage applications, where the particular temperature at which heat is stored may vary across the attainable range of eutectic temperatures. During this work, talc was identified as an inert and stable nucleation particle (NP) that can dramatically increase the observed nucleation rate (thereby decreasing undercooling) in all investigated ZNH pseudobinary eutectics.

The implementation of eutectic salt hydrates into TES applications has been impeded because of concerns of metastability due to (1) their susceptibility to undercool at high degrees and (2) the potential for phase segregation to accompany metastable solidification in cases where different phases exhibit different degrees of undercooling. In the ZNH-based eutectic systems investigated in this study, while large degrees of undercooling are observed, solidification appears to occur simultaneously for both solid phases. In the case in which an NP is added to promote nucleation in the primary phase (ZNH), solidification of both crystalline solids still appears to occur simultaneously, at small degrees of undercooling. Accordingly, there is no evidence for phase segregation or other forms of degradation to occur under the investigated conditions. Thus, the ZNH-based pseudobinary eutectics investigated in this paper are dissimilar from the previously investigated lithium-nitrate-trihydrate-based eutectics, which do demonstrate solidification at different times, and which may therefore be susceptible to degradation caused by phase segregation.^8^

This ZNH pseudobinary eutectics described in this study merit further investigation, in particular in combination with additives to stabilize the shape of the salt hydrates, which can alleviate concerns surrounding volume expansion and cycling in smaller temperature ranges. In particular, one potentially promising method to mitigate these issues is to combine the salt hydrates with small concentrations of gel-forming polymers and thereby exploit the stabilization provided by the resulting polymer network. Additionally, the long-term stability of salt hydrate pseudobinary eutectic PCMs should be evaluated at smaller temperature ranges near the working conditions of TES media for a particular application. In particular, conditions in which the system exhibits extended periods of phase coexistence could introduce additional vectors for instability and degradation of the system over time.

## Supporting Information Available

Polarized light microscopy images of ZNH to investigate melting transformation; supporting calorimetry data; calculations of kinetic parameters from isothermal nucleation rate observations (PDF)
