# Supplementary material for: Zinc Nitrate Hexahydrate Pseudobinary Eutectics for Near-Room-Temperature Thermal Energy Storage
Source: ACS Appl Eng Mater. 2023 Dec 20;2(3):530–41. doi: 10.1021/acsaenm.3c00444 (PMC10964232; doi:10.1021/acsaenm.3c00444)
Supplement: Supplementary file 1 — em3c00444_si_001.pdf [file em3c00444_si_001.pdf]

Supporting Information: Zinc Nitrate Hexahydrate Pseudo-binary Eutectics for Near-Room Temperature Thermal Energy Storage

Sophia Ahmed[1], Denali Ibbotson[1], Chase Somodi[1], Patrick J. Shamberger[1]

[1] Department of Materials Science and Engineering

Texas A&M University

College Station, TX, 77843, USA

Phone: 979-458-1086

Fax: 979-862-6835

E-mail: patrick.shamberger@tamu.edu

SI 1. Variability in ZNH Melting Peaks Between Samples

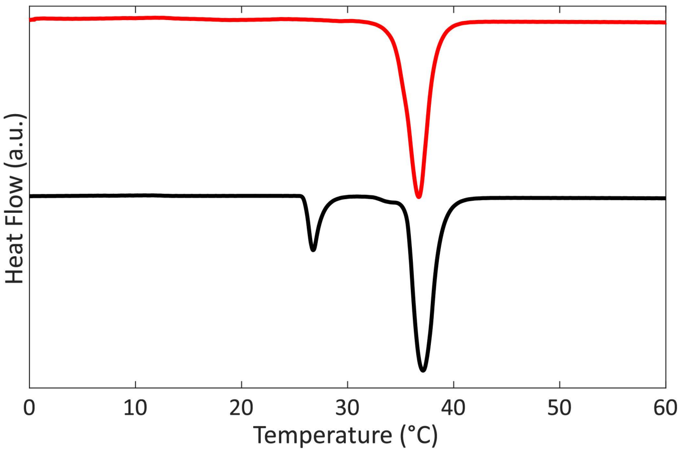

SI Figure 1 DSC scans of two separate prepared samples of as-received 99.998% metals basis ZNH upon heating to evaluate the incipient melting peak observed on DSC. The red line indicated a single melting peak, whereas the black line indicated two melting peaks, suggesting excess water in the sample.

Separate samples of high-purity ZNH, all run at 1 °C·min^{-1}, were placed in the sample holder and backfilled with N_2. Some samples displayed unimodal behavior, and one showed bimodal despite extra cautions being taken to ensure purity of the ZNH. This indicates that there is somewhat stochastic behavior of a melt sample in terms of its melting shape despite all caution being taken to maintain the sample's purity.

SI 2. Solid-Liquid Transformation of ZNH

A thin film of ZNH was heated at 1 °C·min^{-1}, and the transformation associated with the minor DSC peak and the main melting peak (29 to 37 °C temperature range), initially observed with DSC were observed using transmission polarized optical microscopy (SI Figure 2). In 1 °C increments between photos, crystal boundaries are observed to begin melting during the initial peak below the main melting peak, at approximately 32 to 33 °C, indicating that this initial peak indicates incipient melting. No sign of solid-solid transformation is observed. This behavior is consistent with slightly excess $Zn(NO_3)_2$ present in the salt hydrate from the purification process, and initial melting occurring at the $Zn(NO_3)_2 \cdot 6(H_2O)$–$Zn(NO_3)_2 \cdot 4(H_2O)$ eutectic point.

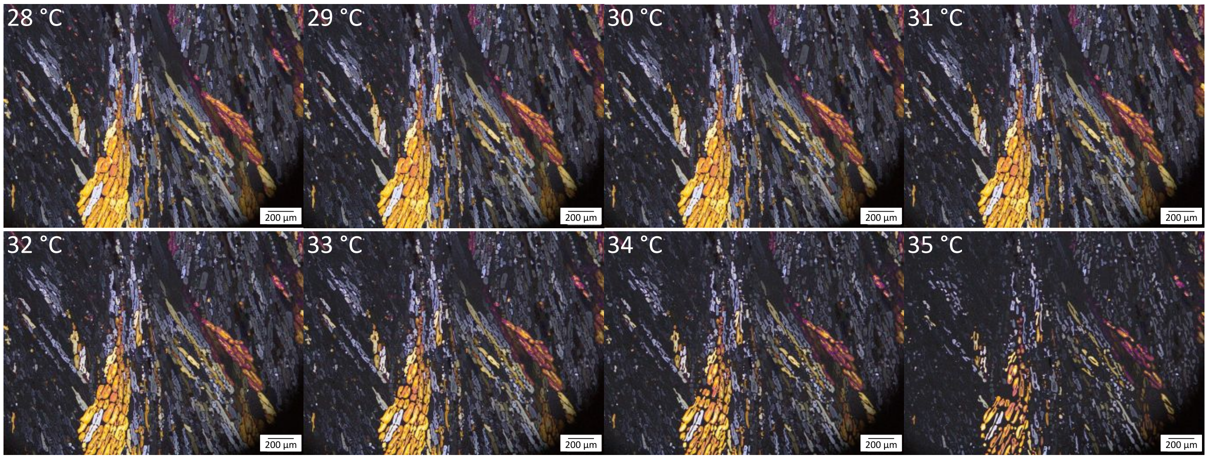

SI Figure 2 Polarized light microscopy of ZNH upon heating to evaluate the incipient melting peak observed on DSC.

SI 3. Evaluation of Onset and Peak Temperatures at Various Ramp Rates

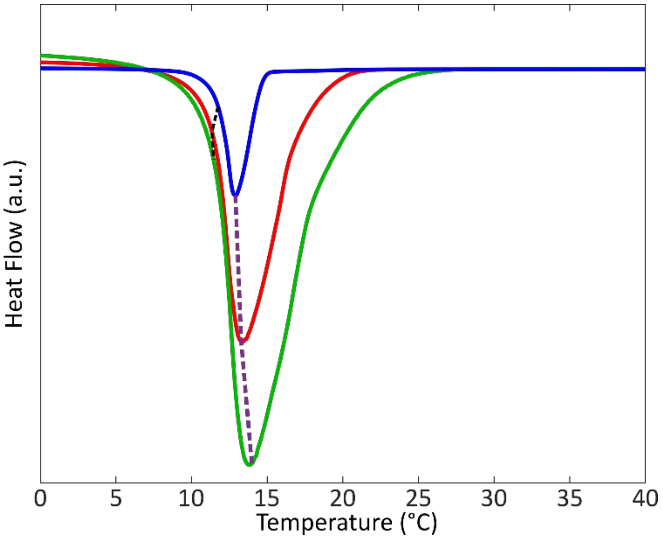

SI Figure 3 DSC curves highlighting different ramp rates with a black dashed line connecting onset temperatures and a purple dashed line connecting peak temperatures of the melting curve. The blue, red, and green lines represent the 1, 5, and 10 °C·min^{-1} ramp rates respectively.

To evaluate the use of the onset temperature as the rate-insensitive temperature, as expected for an invariant eutectic transformation, one pan of ZNH-NH_4NO_3 was scanned using the Q2000 DSC at various ramp rates (1 to 10 °C·min^{-1}). As heating ramp rates increased, the onset temperature deviated by < 0.5 °C, whereas the peak temperature deviated by nearly 1 °C (SI Table 1), with the faster ramp rates indicating a higher temperature melting peak, as expected due to internal temperature gradients within the sample volume. This indicates that the onset temperature is the more reliable value for a eutectic temperature.

SI Table 1. Onset and peak temperatures of ZNH-NH_4NO_3 at different ramp rates

Ramp Rate	T_{onset}	T_{peak}
°C·min^{-1}	°C	°C
1	11.74	12.88
5	11.34	13.33
10	11.44	13.85

SI 4. X-Ray Diffraction of As-Received vs Dried Talc

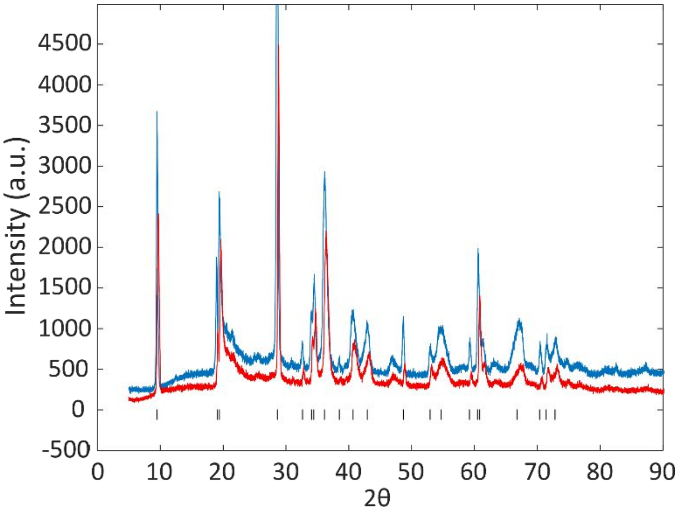

SI Figure 4 X-ray diffraction pattern of as-received talc in blue and talc dried at 200 °C in red, with peaks indicated by tick marks on the bottom.

As-received talc and dried talc were scanned using powder-diffraction XRD to see if the drying process altered the structure of the talc (SI Figure 3). The drying process was applied to remove excess moisture which can lead to particle agglomeration and can change the water concentration of the salt hydrate. There was minimal difference between both patterns, indicating that heating the talc to 200 °C did not alter the structure.

SI 5. Nucleation Time per Degree of Undercooling Derivations

Cumulative distribution functions (CDFs) were compiled from repeated isothermal testing, from which the characteristic times associated with the 25th, 50th, and 75th percentiles ($\tau_{0.25}\tau$,$_{0.50}\tau$,$_{0.75}$) are determined (SI Figures 5,7,9,11), where red indicates samples with talc and blue is neat samples. The fit for $\beta_{0.50}$ is included for both cases, along with the uncertainty bounds of the prediction of the model. From these characteristic times, characteristic rates ($1/\tau_{0.25}$, $1/\tau_{0.50}$, $1/\tau_{0.75}$) are determined (SI Figures 6,8,10,12), and

are used to fit nucleation rates $\beta_P = a\Delta T^b$, where P = 0.25, 0.5, 0.75. Parameters a and b are reported in Table 7 in the main text.

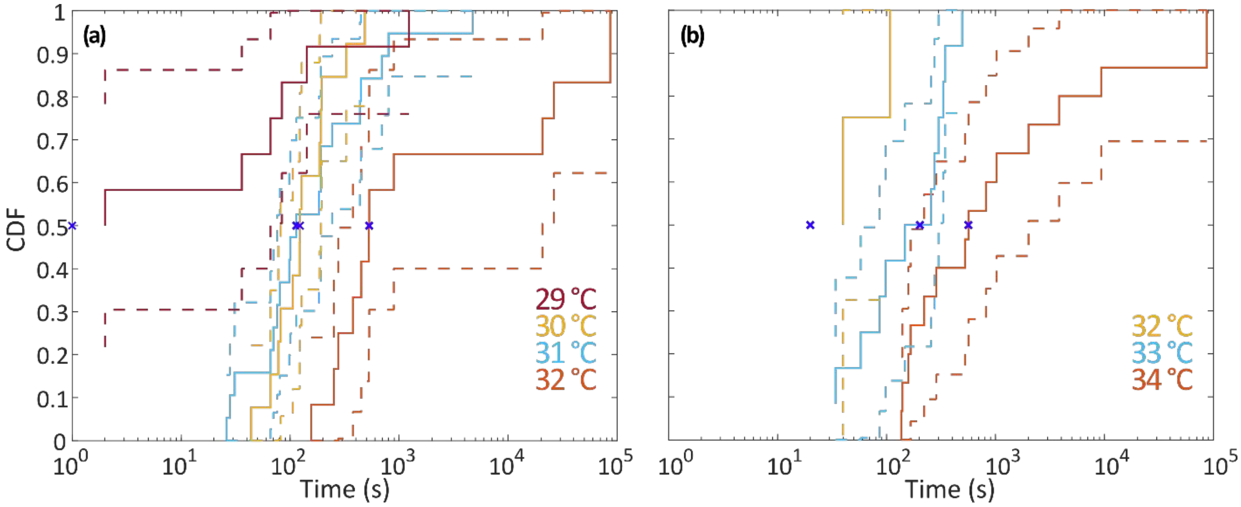

SI Figure 5 Cumulative distribution function (CDF) plots at various temperatures at various temperatures for ZNH, for (a) pure ZNH and (b) ZNH including 2 wt% talc. Colors represent isothermal temperature, as indicated. Dark blue x's represent the median values ($\tau_{0.50}$). Solid lines represent the CDF of the collected data, and dotted lines represent the lower and upper bounds of the 95% confidence interval of the data collected at each temperature.

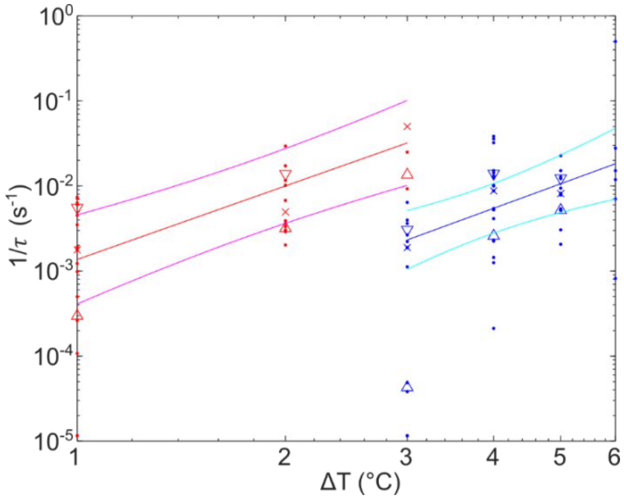

SI Figure 6 Characteristic rates of ZNH ($1/\tau$, dots). $1/\tau_{0.25}$, $1/\tau_{0.50}$, $1/\tau_{0.75}$ are represented by the upwards triangle, x, and downwards triangle, respectively. Solid lines represent fit to $1/\tau_{0.50}$, with upper and lower prediction bounds on the model. A red central line indicates samples with talc, and blue indicates neat.

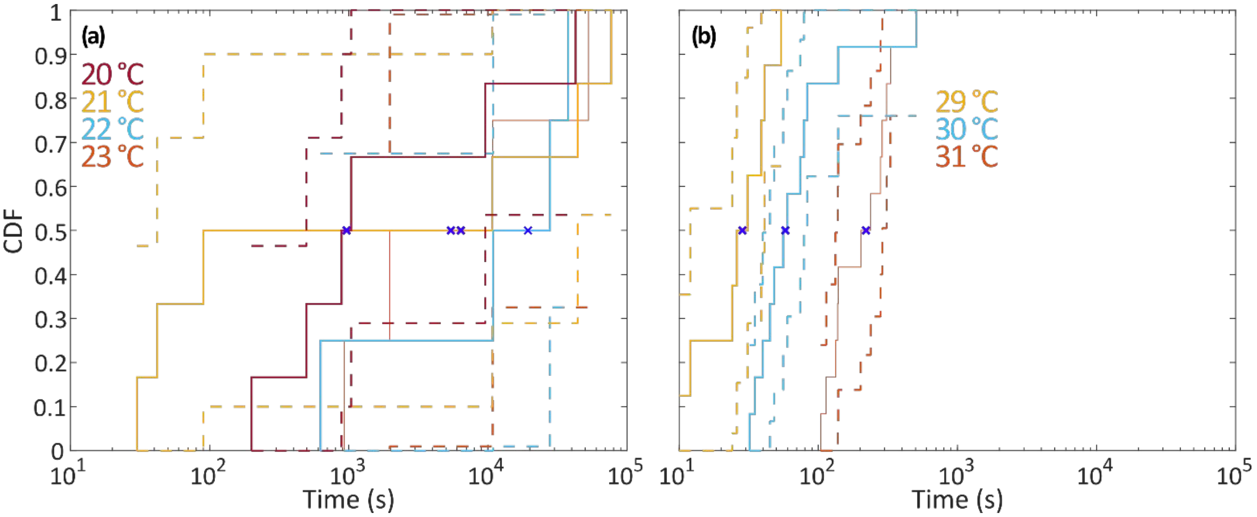

SI Figure 7 Cumulative distribution function (CDF) plots at various temperatures at various temperatures for ZNH-$NaNO_3$, for (a) ZNH-$NaNO_3$, and (b) ZNH-$NaNO_3$, including 2 wt% talc. Colors represent isothermal temperature, as indicated. Dark blue x's represent the median values ($\tau_{0.50}$). Solid lines represent the CDF of the collected data, and dotted lines represent the lower and upper bounds of the 95% confidence interval of the data collected at each temperature.

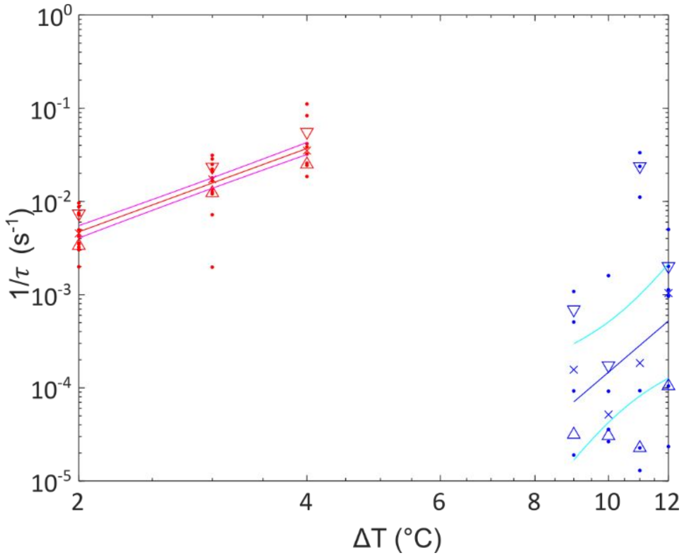

SI Figure 8 Characteristic rates of ZNH-$NaNO_3$ ($1/\tau$, dots). $1/\tau_{0.25}$, $1/\tau_{0.50}$, $1/\tau_{0.75}$ are represented by the upwards triangle, x, and downwards triangle, respectively. Solid lines represent fit to $1/\tau_{0.50}$, with upper and lower prediction bounds on the model. A red central line indicates samples with talc, and blue indicates neat.

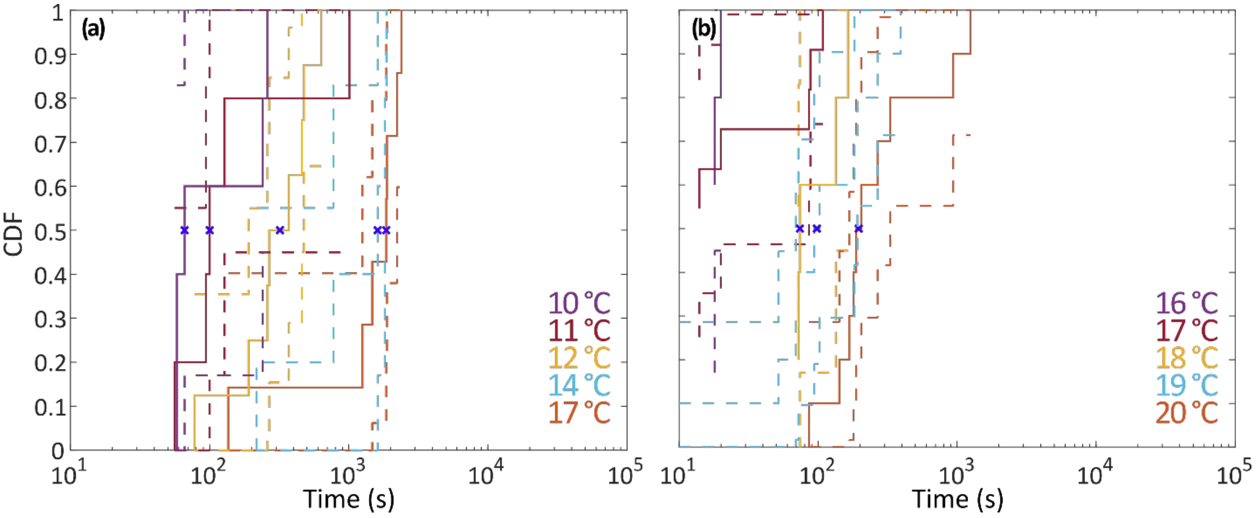

SI Figure 9 Cumulative distribution function (CDF) plots at various temperatures at various temperatures for ZNH-KNO_3, for (a) ZNH-KNO_3, and (b) ZNH-KNO_3, including 2 wt% talc. Colors represent isothermal temperature, as indicated. Dark blue x's represent the median values ($\tau_{0.50}$). Solid lines represent the CDF of the collected data, and dotted lines represent the lower and upper bounds of the 95% confidence interval of the data collected at each temperature.

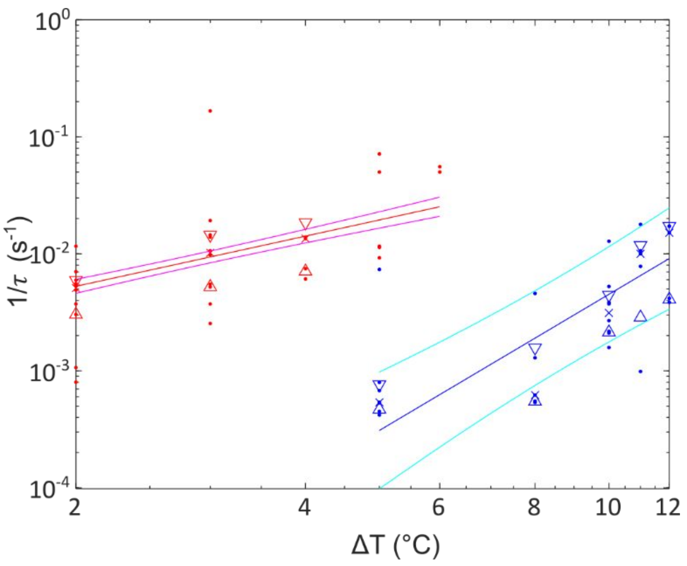

SI Figure 10 Characteristic rates of ZNH-KNO_3 ($1/\tau$, dots). $1/\tau_{0.25}$, $1/\tau_{0.50}$, $1/\tau_{0.75}$ are represented by the upwards triangle, x, and downwards triangle, respectively. Solid lines represent fit to $1/\tau_{0.50}$, with upper and lower prediction bounds on the model. A red central line indicates samples with talc, and blue indicates neat.

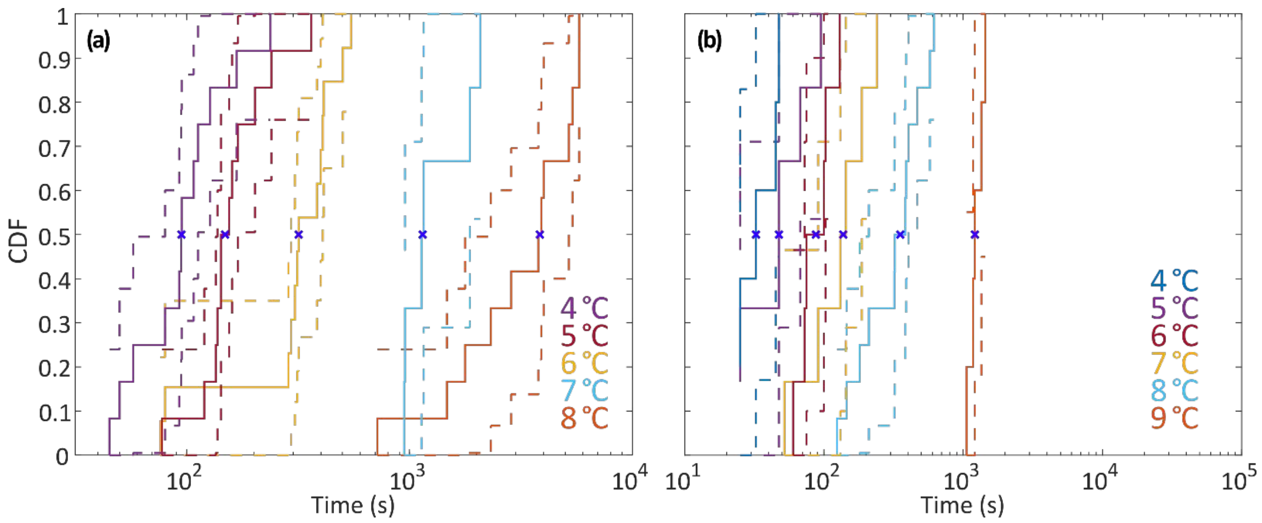

SI Figure 11 Cumulative distribution function (CDF) plots at various temperatures at various temperatures for ZNH-NH_4NO_3, for (a) ZNH-NH_4NO_3, and (b) ZNH-NH_4NO_3, including 2 wt% talc. Colors represent isothermal temperature, as indicated. Dark blue x's represent the median values ($\tau_{0.50}$). Solid lines represent the CDF of the collected data, and dotted lines represent the lower and upper bounds of the 95% confidence interval of the data collected at each temperature.

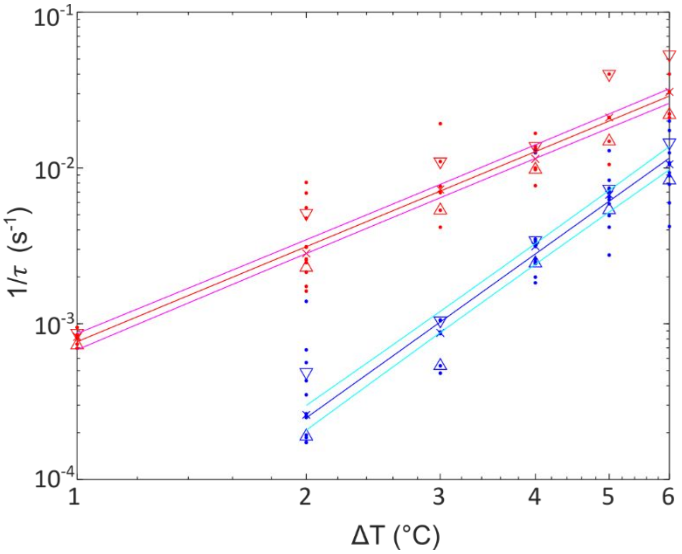

SI Figure 12 Characteristic rates of ZNH-NH_4NO_3 ($1/\tau$, dots). $1/\tau_{0.25}$, $1/\tau_{0.50}$, $1/\tau_{0.75}$ are represented by the upwards triangle, x, and downwards triangle, respectively. Solid lines represent fit to $1/\tau_{0.50}$, with upper and lower prediction bounds on the model. A red central line indicates samples with talc, and blue indicates neat.

SI 6. Derived Slopes for Trendlines of Figure 10

The slopes (and their variance) describing the cycling dependence in thermophysical characteristics are illustrated in Figure 10 in the main text (SI Table 2). The high variance in undercooling found in the neat values for ZNH, ZNH-$NaNO_3$, and ZNH-KNO_3, highlighted in red underlined text, is attributable to the increasing degree of undercooling with cycling in these systems.

SI Table 2. Slopes and uncertainties of fitted trendlines for Figure 10

Sample			Integrated Melting Area		Melting Temperature		Degree of Undercooling	
			$\frac{d}{dN}$	2σ	$\frac{d°C}{dN}$	2σ	$\frac{d°C}{dN}$	2σ
ZNH	Talc	A	-7.2×10^{-4}	$1.5\text{x}10^{-4}$	-1.3×10^{-3}	$1.9\text{x}10^{-4}$	2.6×10^{-3}	$7.6\text{x}10^{-4}$
		B	-4.9×10^{-3}	$2.9\text{x}10^{-4}$	2.1×10^{-3}	$3.2\text{x}10^{-4}$	-4.5×10^{-3}	$7.7\text{x}10^{-4}$
	Neat	A	-4.1×10^{-4}	$1.8\text{x}10^{-4}$	-4.9×10^{-4}	$1.9\text{x}10^{-4}$	3.1×10^{-2}	$4.4\text{x}10^{-3}$
		B	-1.7×10^{-3}	$2.1\text{x}10^{-4}$	-1.3×10^{-4}	$1.1\text{x}10^{-4}$	6.5×10^{-2}	$4.1\text{x}10^{-3}$
ZNH-$NaNO_3$	Talc	A	-2.0×10^{-3}	$1.0\text{x}10^{-4}$	-4.7×10^{-3}	$2.6\text{x}10^{-4}$	4.6×10^{-3}	$6.9\text{x}10^{-4}$
		B	-1.9×10^{-3}	$1.3\text{x}10^{-4}$	-4.4×10^{-3}	$6.4\text{x}10^{-4}$	4.4×10^{-3}	$8.0\text{x}10^{-4}$
	Neat	A	1.4×10^{-5}	$1.3\text{x}10^{-4}$	-3.3×10^{-4}	$2.9\text{x}10^{-4}$	3.9×10^{-2}	$5.9\text{x}10^{-3}$
		B	-1.0×10^{-3}	$1.5\text{x}10^{-4}$	-6.4×10^{-4}	$2.9\text{x}10^{-4}$	6.4×10^{-2}	$9.3\text{x}10^{-3}$
ZNH-KNO_3	Talc	A	4.3×10^{-6}	$1.9\text{x}10^{-4}$	-3.6×10^{-4}	$3.5\text{x}10^{-4}$	6.5×10^{-3}	$1.2\text{x}10^{-3}$
		B	2.1×10^{-4}	$2.4\text{x}10^{-4}$	-1.3×10^{-3}	$5.3\text{x}10^{-4}$	-4.6×10^{-3}	$1.2\text{x}10^{-3}$
	Neat	A	4.3×10^{-4}	$2.1\text{x}10^{-4}$	-1.3×10^{-3}	$5.7\text{x}10^{-4}$	5.1×10^{-2}	$3.6\text{x}10^{-3}$
		B	1.2×10^{-3}	$2.1\text{x}10^{-4}$	7.1×10^{-4}	$4.6\text{x}10^{-4}$	3.5×10^{-2}	$4.7\text{x}10^{-3}$
ZNH-NH_4NO_3	Talc	A	-6.0×10^{-4}	$1.8\text{x}10^{-4}$	-2.6×10^{-3}	$4.1\text{x}10^{-4}$	3.1×10^{-3}	$1.1\text{x}10^{-3}$
		B	-1.9×10^{-3}	$1.7\text{x}10^{-4}$	-4.3×10^{-3}	$9.9.\text{x}10^{-4}$	1.3×10^{-3}	$1.5\text{x}10^{-3}$
	Neat	A	1.8×10^{-3}	$2.3\text{x}10^{-4}$	-2.9×10^{-3}	$4.5\text{x}10^{-4}$	-8.4×10^{-5}	$6.9\text{x}10^{-4}$
		B	3.5×10^{-4}	$1.7\text{x}10^{-4}$	2.2×10^{-3}	$8.9\text{x}10^{-4}$	-6.3×10^{-4}	$9.5\text{x}10^{-4}$
